# PINK1 deficiency enhances alveolar regeneration and basement membrane remodeling by reducing YAP phosphorylation during lung ischemia-reperfusion injury

**DOI:** 10.1038/s41419-026-09149-3

**Published:** 2026-08-05

**Authors:** Yifan Yu, Xiaolong Hu, Jinsheng Li, Jinghao Huang, Zhiheng Ji, Qiuyu Fang, Yongchao Wang, Zhiling Lou, Weiping Zhang, Fuxiang Liang, Ming Wu

**Affiliations:** 1 Department of Thoracic Surgery, The Second Affiliated Hospital, Zhejiang University School of Medicine, Hangzhou, China; 2 Department of Pharmacology, School of Basic Medical Sciences, Zhejiang University, Hangzhou, China

**Keywords:** Respiratory tract diseases, Kinases

## Abstract

Alveolar repair and regeneration are critical for resolving lung ischemia-reperfusion injury (LIRI); however, the molecular mechanisms that restrain this process remain unclear. Here, we identify PTEN-induced kinase 1 (PINK1) as a critical regulator of alveolar regeneration via Hippo-YAP signaling in LIRI. Wild-type and *Pink1*^*-/-*^ mice, as well as mice with adeno-associated virus type 6-mediated alveolar epithelium-specific knockdown or overexpression of PINK1, were subjected to left lung ischemia-reperfusion. In vitro, murine alveolar epithelial MLE-12 cells were exposed to oxygen-glucose deprivation/recovery (OGD/R) with PINK1 modulated genetically. We demonstrated that PINK1 deficiency markedly enhanced, while PINK1 overexpression attenuated, type II alveolar epithelial cell (AT2) proliferation, nuclear translocation of YAP, and the expression of YAP target genes in LIRI. Pharmacologic inhibition of YAP abrogated, whereas YAP activation potentiated, the pro-regenerative phenotype induced by OGD/R under PINK1 knockdown. Mechanistically, PINK1 directly phosphorylated YAP at Ser127, promoting YAP cytoplasmic retention and stabilization. PINK1 deficiency reduced YAP phosphorylation, leading to increased nuclear translocation of YAP and enhanced regenerative signaling. Meanwhile, PINK1 deficiency induced alveolar basement membrane thickening and lung function decline. Collectively, our findings reveal PINK1 as a kinase-dependent brake on YAP-driven alveolar regeneration. However, PINK1 deficiency facilitates alveolar repair while simultaneously precipitating maladaptive remodeling and functional deterioration in murine LIRI, suggesting that balanced regulation of the PINK1-YAP pathway is essential during LIRI repair.

## Introduction

Lung ischemia-reperfusion injury (LIRI) commonly occurs in clinical practices such as lung transplantation [1, 2], extracorporeal circulation [3], resuscitation after severe hemorrhagic shock [4], and pulmonary embolism reperfusion [5]. In particular, during lung transplantation, LIRI is the primary cause of primary graft dysfunction (PGD) and serves as a critical determinant of post-transplant prognosis [1, 6]. Despite its clinical significance, specific and effective therapeutic strategies for LIRI remain unavailable due to a poor understanding of its complex pathophysiological mechanisms [7].

Following injury, endogenous repair programs are activated to restore homeostasis, since the lung retains a certain regenerative capacity [8]. A key step of the repair programs is the regeneration of the alveolar epithelial cell layer [9], an essential component of the alveolar-capillary barrier which consists of alveolar epithelial cell layer and capillary endothelial cell layer separated by a thin basement membrane to facilitate gas exchange. The alveolar epithelial cell layer comprises two principal cell types, alveolar type I (AT1) and type II (AT2) epithelial cells [10, 11]. AT1 cells are specialized for gas exchange [12], whereas AT2 cells are traditionally known for synthesizing and secreting pulmonary surfactant to reduce alveolar surface tension [13]. In LIRI, AT1 cells loss extensively due to their highly vulnerable to insults, leading to increased barrier permeability, and alveolar flooding [14]. More recently, AT2 cells have been firmly established as the resident progenitor cell population of the distal lung, capable of rapidly proliferating and differentiating into AT1 cells, thereby driving structural reconstruction and functional recovery after injury [15, 16]. The AT2‑to‑AT1 intermediates are indispensable for proper AT1 differentiation [17], as differentiated epithelial cells produce matrix components necessary for re-establishing epithelial-basement membrane interactions [18]. The structural integrity of the alveolar basement membrane directly determines the efficiency of gas exchange [18]. Thus, basement membrane remodeling in LIRI protects lung tissue function and alleviates lung injury [19]. Yet, when re-epithelialization is dysregulated, it may contribute to maladaptive remodeling [20, 21].

The Hippo-YAP signaling pathway plays a pivotal role in physiological alveolar regeneration [14]. Its core components include mammalian sterile 20-like kinase 1/2 (MST1/2), large tumor suppressor 1/2 (LATS1/2), and Yes-associated protein (YAP) or transcriptional coactivator with PDZ-binding motif (TAZ) [22]. Activation of Hippo signaling triggers sequential phosphorylation of YAP/TAZ, leading to their cytoplasmic retention and subsequent degradation [23]. Conversely, pathway inhibition prevents phosphorylation, allowing YAP/TAZ nuclear translocation to drive the transcription of pro-proliferative genes [24, 25]. Although this pathway is recognized as a regulator of tissue regeneration, its precise involvement in LIRI-associated alveolar repair remains unclear.

Of particular interest to us is a single-cell transcriptomic profiling study of ischemia-reperfusion responses in human lung transplants [26], which showed that AT2 cells expressing YAP also expressed PTEN-induced kinase 1 (PINK1), a serine/threonine kinase best known for initiating mitophagy *via* the PINK1-Parkin pathway [27, 28]. PINK1 attenuates ischemia-reperfusion injury in the heart, liver, kidney, and brain through the induction of mitophagy [29–32]. However, PINK1 is not exclusively localized to mitochondria; it is also detected in the cytosol [33], where it functions as a pleiotropic kinase capable of regulating diverse cellular processes independent of mitophagy [34]. Beyond its role in mitochondrial quality control, PINK1 was originally identified as a putative tumor suppressor [35, 36], and its expression is frequently downregulated across various malignancies [35]. Moreover, PINK1 deficiency promotes fibrotic remodeling in the aging lung [37] and sustains cell proliferation by reprogramming glucose metabolism via HIF-1α stabilization in cultured mouse embryonic fibroblasts and primary cortical neurons [35]. Collectively, these findings implicate PINK1 as a multifaceted regulator of both cell proliferation and tissue regeneration. Yet, whether PINK1 regulates alveolar epithelial and alveolar basement membrane regeneration following LIRI remains unknown.

In this study, we demonstrate that PINK1 regulates YAP phosphorylation and that PINK1 deficiency enhances alveolar regeneration and basement membrane remodeling by reducing YAP phosphorylation during LIRI in mice. Our findings provide mechanistic insights into imperfect alveolar regeneration following LIRI and reveal key aspects of its complex pathophysiology. Furthermore, the results suggest a potential therapeutic strategy aimed at improving outcomes in LIRI.

## Materials and Methods

See the online supplement for detailed methods.

## Results

### PINK1 deficiency upregulates the Hippo-YAP pathway in mice lung

Analysis of single-cell datasets from human lung transplants [26] revealed that AT2 cells expressing *YAP1* also express *PINK1* during lung ischemia-reperfusion injury (Fig. S1A–C), suggesting a potential link between PINK1 and the Hippo-YAP pathway. To explore the function of PINK1 in LIRI and clarify its effect on alveolar epithelial regeneration post-LIRI, *Pink1* knockout (*Pink1*^*-/-*^) mice [38] were utilized.

**Fig. 1:**
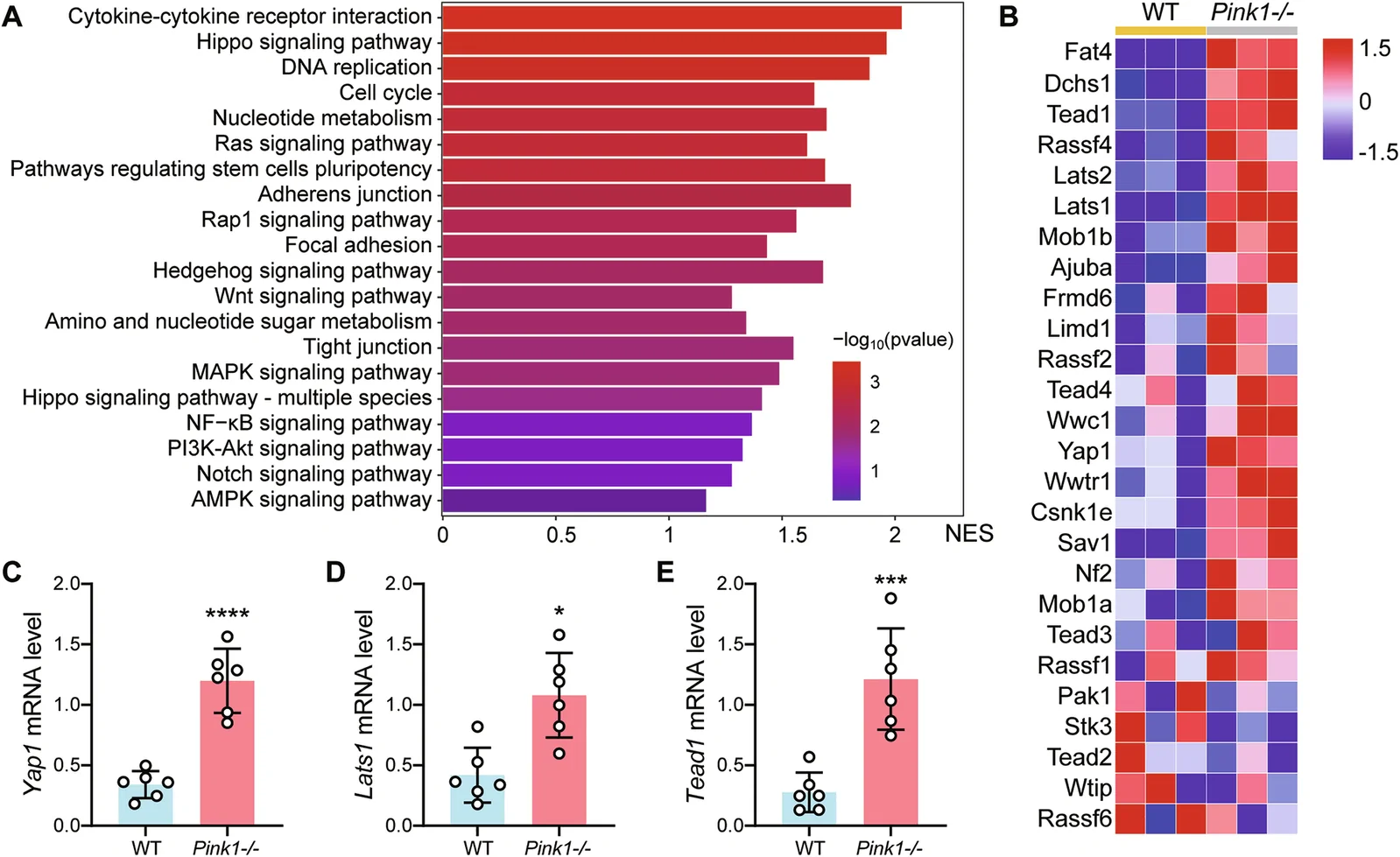
PINK1 deficiency upregulates the Hippo-YAP pathway in mouse lung tissue. **A** Gene Set Enrichment Analysis (GSEA) of the top 20 enriched pathways related to proliferation, regeneration, and repair; **B** Heatmap of the expression levels of different molecules in the Hippo-YAP signaling pathway; qRT-PCR analysis of *Yap1* (**C**), *Lats1* (**D**) and *Tead1* (**E**) mRNA transcription levels. Data are presented as mean ± SD. Compared to the wild-type group (WT): *p < 0.05, **p < 0.01, ***p < 0.001, ****p < 0.0001; *t*-test. (*Pink1-/-*: PINK1 knockout group; NES: Normalized enrichment score).

We first profiled the transcriptomic differences between wild-type (WT) and *Pink1*^*-/-*^ mice using lung tissue. Through KEGG and GSEA pathway analyses - focused on the pathways linked to injury, proliferation, and repair (Fig. 1A), we identified a striking upregulation of the Hippo-YAP signaling pathway in *Pink1*^*-/-*^ lungs.

Further interrogation via heatmap visualization confirmed significant upregulation of core regulatory components of the Hippo-YAP pathway, including LATS, YAP, and TEAD (Fig. 1B). These findings were validated by RT-qPCR, which demonstrated concordant upregulation of *Lats1, Yap1, and Tead1* mRNA levels in *Pink1*^*-/-*^ lungs (Fig. 1C–E).

Given that the Hippo-YAP pathway is a well-established driver of endogenous alveolar epithelial regeneration following lung injury [15, 39], our transcriptomic data suggest a novel link: PINK1 deficiency enhances Hippo-YAP pathway activity. Collectively, these results position PINK1 as a potential regulator of alveolar epithelial repair and alveolar regeneration, likely acting through the Hippo-YAP pathway during lung injury. Thus, we suppose that PINK1 regulates alveolar regeneration via Hippo-YAP signaling in the context of lung injury.

### Characterizing alveolar epithelial regeneration after LIRI in mice

To delineate the timeline and key determinants of alveolar epithelial regeneration following LIRI, we evaluated lung injury and regeneration in mice at multiple post-injury time points. A lung ischemia/reperfusion (I/R) model was employed to recapitulate the LIRI process: a left pulmonary hilar clamping for 1 hour to mimic lung ischemia, and the clip was then released to simulate reperfusion in mice. Six distinct reperfusion durations are indicated: 0 h, 6 h, 24 h, 72 h, 7 days, and 14 days (Fig. S1D).

**Fig. 2:**
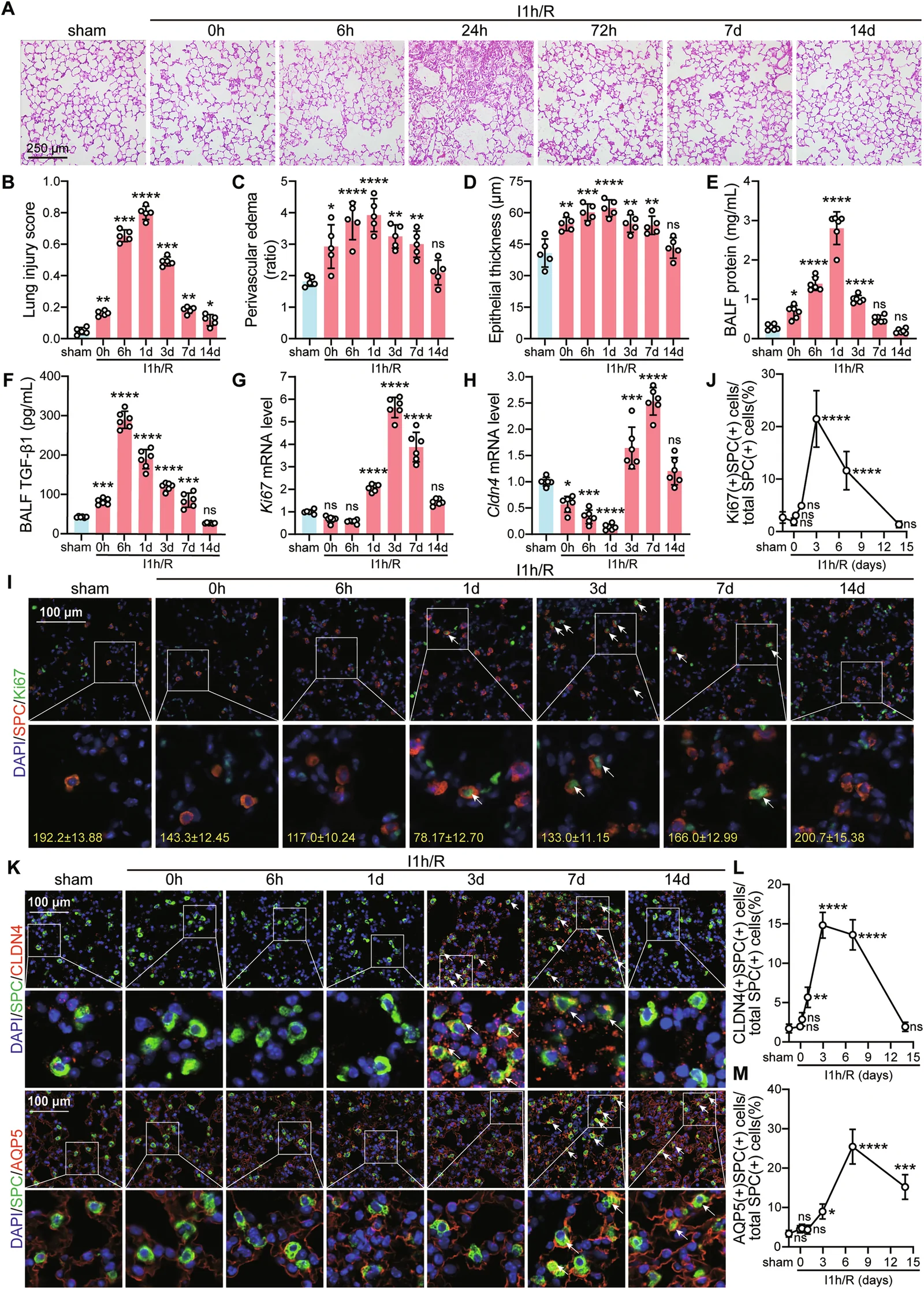
Dynamic changes in lung injury and regeneration after ischemia-reperfusion in mice. Mice were subjected to 1 h of left lung ischemia followed by reperfusion for the indicated times (I 1 h/R). **A** Representative HE staining of left lung sections at different reperfusion time points. **B** Lung injury score of the left lung. **C** Perivascular edema index of the left lung. **D** Airway wall thickness of the left lung. **E** Total protein concentration in bronchoalveolar lavage fluid (BALF) from the left lung. **F** TGF-β1 concentration in BALF. **G**, **H** Relative mRNA expression of *Ki67* and *Cldn4* in the left lung. **I** Representative immunofluorescence (IF) co-staining of SPC (red, AT2 cell marker) and Ki67 (green) in the left lung. White arrows indicate SPC^+^Ki67^+^ cells. Yellow text in the left corner indicates the quantification of SPC^+^ cells per field. **J** Quantification of the percentage of Ki67^+^SPC^+^ cells among total SPC^+^ cells. **K** Representative immunofluorescence co-staining of SPC (green) with CLDN4 (red) or AQP5 (red)in the left lung. White arrows indicate SPC^+^CLDN4^+^ cells or SPC^+^AQP5^+^cells. Quantification of the percentage of SPC⁺CLDN4⁺ (**L**) and SPC⁺AQP5⁺ (**M**) cells among total SPC⁺ cells. Data are presented as mean ± SD. Compared with sham group: *p < 0.05, **p < 0.01, ***p < 0.001, ****p < 0.0001; ns, not significant; One-way ANOVA.

Histopathological assessment by HE staining of the left lung showed that at 24 h post-I/R marked the peak of injury, the alveolar septa were maximally thickened, hyaline membrane formation was most prominent, and neutrophil infiltration peaked, indicating the most severe lung injury at this time point. By day 7 post-I/R, the lung tissue architecture had largely recovered, resembling that of sham-operated mice (Fig. 2A). The time course for lung injury score, vascular edema, airway wall thickness, and total protein concentration in bronchoalveolar lavage fluid (BALF) exhibited a consistent chronological pattern of change, mirroring the histological findings (Fig. 2B–E).

Since anti-inflammatory factors such as transforming growth factor-β1 (TGF-β1) and interleukin-10 (IL-10) are established harbingers of tissue repair initiation [40, 41], we measured the concentrations of TGF-β1 and IL-10 in BALF to estimate the approximate onset time of lung tissue regeneration. TGF-β1 levels surged at 6 hours post-I/R before declining (Fig. 2F), suggesting that lung repair and regeneration processes commence as early as 6 hours of reperfusion. In contrast, IL-10 levels increased immediately after reperfusion but rapidly returned to sham levels (Fig. S1E), indicating a transient early anti-inflammatory response.

Alveolar type II epithelial cells (AT2 cells) function as alveolar stem cells in the adult lung, capable of rapid self-renewal and differentiation into AT1 cells after lung injury [15]. Analysis of *Ki67* transcription, an indicator of cellular proliferation, showed a gradual decline early in reperfusion, a rebound between 6 and 24 hours, and a peak at day 3, followed by a progressive decline, suggesting that day 3 post-I/R corresponds to the height of regenerative proliferation (Fig. 2G). We then examined CLDN4 expression [42], a marker of intermediate AT2-to-AT1 transition states (Fig. 2H). The results showed that AT2 cell differentiation (spanning day1-3) occurred after the onset of proliferation and peaked around day 7 post-I/R.

To directly visualize alveolar regeneration dynamics, we performed immunofluorescence (IF) co-staining of SPC (surfactant protein C, an AT2 marker) and Ki67. The proportion of SPC and Ki67 double-positive (SPC^+^ Ki67^+^) cells among all SPC-positive cells was highest at day 3 post-I/R (Fig. 2I, J), indicating that AT2 proliferation also peaked at this time point. We also performed IF co-staining of SPC and CLDN4 together with SPC and AQP5(AT1 cell marker). The results showed that the proportion of SPC^+^CLDN4^+^ cells peaked between days 3 and 7, while the proportion of SPC^+^AQP5^+^ cells peaked on day 7 post-I/R (Fig. 2K–M). The results suggested that AT2-to-AT1 differentiation occurred.

Together, the above results indicated a phased regenerative program after LIRI, including an early anti-inflammatory and proliferation phase (beginning around 6 hours, peaking at day 3 post-I/R) followed by AT2 differentiation and structural restoration (peaking near day 7 post-I/R).

### Dynamic changes of YAP in alveolar epithelial cells during regeneration post‑I/R

**Fig. 3:**
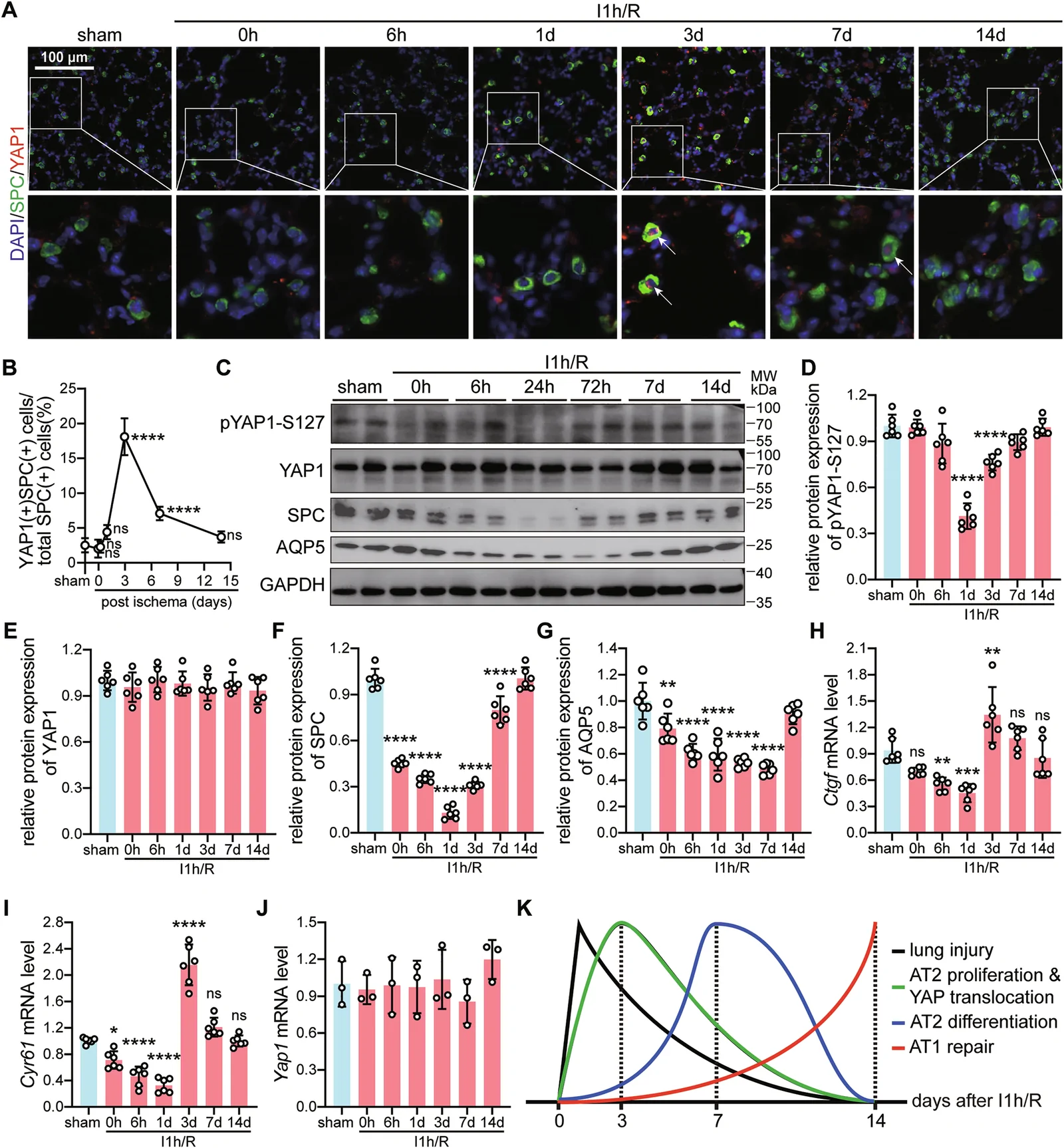
Dynamic changes of YAP signaling in mouse lungs after ischemia-reperfusion. Mice were subjected to 1 hour of left lung ischemia followed by reperfusion for the indicated times (I 1 h/R). **A** Representative immunofluorescence co-staining SPC (green, AT2 cell marker) and YAP1 (red) in the left lung. White arrows indicate SPC^+^YAP1^+^ cells. **B** Quantification of YAP1 nuclear localization among SPC^+^ cells. **C** Representative Western blotting analysis of YAP1, pho-YAP1 (Ser127), SPC, and AQP5 in the left lung. **D**–**G** Quantification of relative protein expression levels of pho-YAP1 (Ser127), YAP1, SPC, and AQP5. **H**–**J** Relative mRNA expression levels of *Ctgf, Cyr61* and *Yap1* in the left lung. (**K**) Schematic illustration summarizing the temporal pattern of lung injury and regeneration after I/R in mice. Data are presented as mean ± SD. Compared with the sham group: *p < 0.05, **p < 0.01, ***p < 0.001, ****p < 0.0001; ns, not significant; One-way ANOVA.

To characterize the dynamic changes of YAP during alveolar epithelial regeneration, we examined its subcellular localization and expression across multiple reperfusion time points. IF co-staining of SPC and YAP1 revealed that at day 3 after I/R, the proportion of SPC and YAP1 double-positive cells, with YAP clearly co-localized with nuclear DAPI, was the highest among all SPC-positive cells, indicating the greatest extent of YAP nuclear translocation in AT2 cells at this time point (Fig. 3A, B). This indicated maximal nuclear translocation of YAP in AT2 cells at this time point, consistent with our earlier observation that day 3 post-I/R coincides with the peak of alveolar epithelial proliferation.

Western blot analysis (Fig. 3C–G) further confirmed that the phosphorylated, inactive form of YAP (pYAP) reached its lowest level at day 1 after I/R, when YAP is primed for nuclear translocation and activation of its pro-proliferative function. In contrast, total YAP protein level showed no significant changes across all time points after I/R, suggesting that lung regeneration post-I/R is regulated by post-translational modification of YAP rather than changes in overall YAP abundance.

This temporal pattern paralleled changes in SPC and AQP5 expression. Phosphorylated-YAP (pYAP) dropped markedly by 24 hours after I/R, marking the initiation of regeneration signaling; SPC levels began rising at day 1 after I/R, reflecting AT2 cell proliferation; and AQP5 levels increased starting at day 3 after I/R, suggesting differentiation of a subset of AT2 cells into AT1 cells (Fig. 3C–G). Following YAP nuclear translocation, the transcription levels of its classic downstream targets, *Ctgf* and *Cyr61*, peaked at day 3 after I/R, with the transcriptional maxima occurring slightly later than the nadir of pYAP (Fig. 3H, I). However, we did not observe any statistical differences in *Yap1* transcription levels across all the time points in the lung tissues (Fig. 3J).

Collectively, the above results delineated the coordinated temporal changes of lung injury severity and regenerative and repair activity after lung ischemia-reperfusion in mice (Fig. 3K). From this comprehensive profile, we identified day 3 after LIRI is an optimal time point for observing lung tissue regeneration and Hippo-YAP signaling activation. Accordingly, all subsequent experiments employed the model with the uniform setting conditions: 1 hour of ischemia followed by 3 days of reperfusion.

### PINK1 deficiency promotes alveolar regeneration after LIRI

**Fig. 4:**
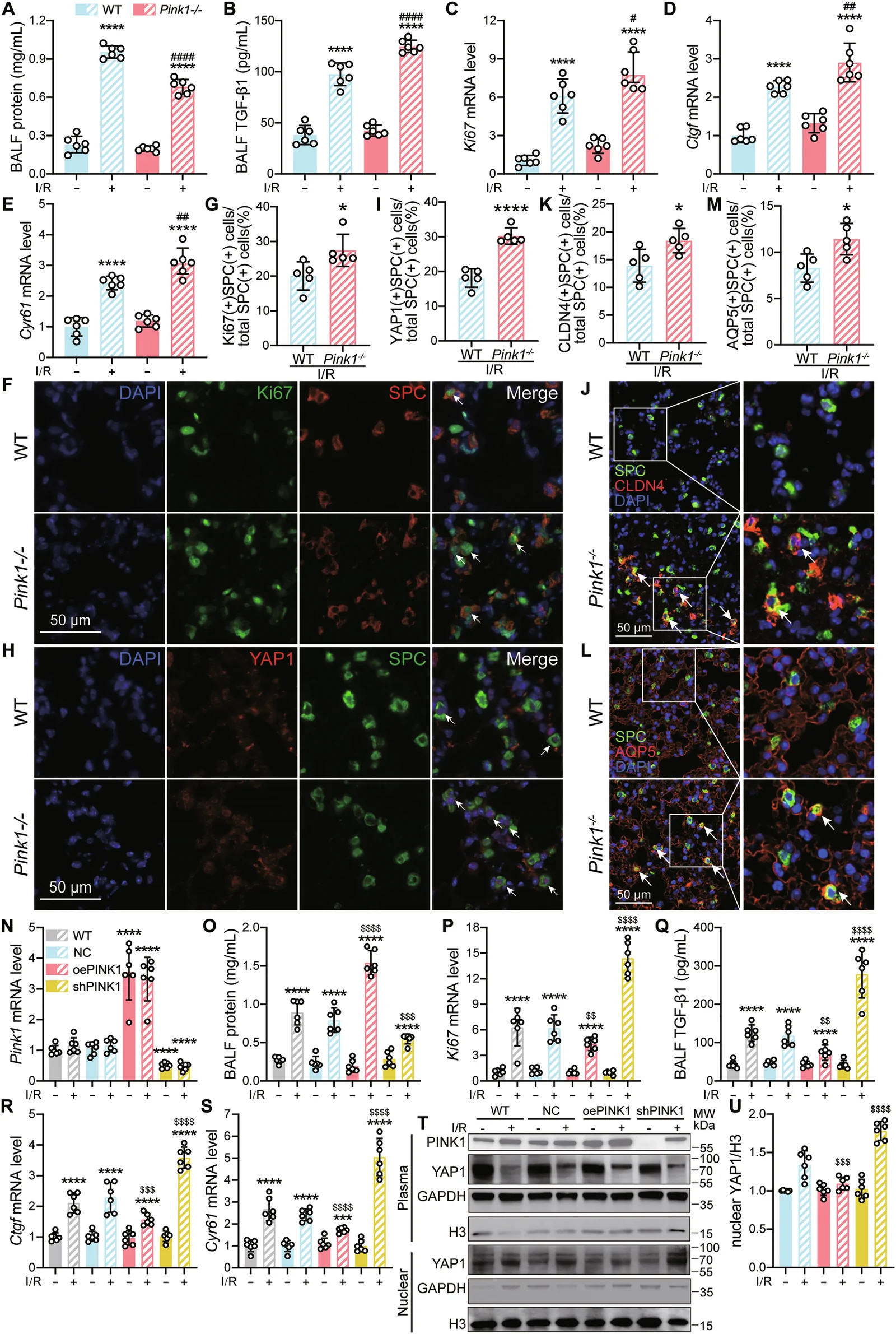
PINK1 deletion or knockdown promotes alveolar regeneration after lung ischemia-reperfusion injury. Mice were subjected to 1 hour of left lung ischemia followed by reperfusion for 3 days (I 1 h/R 3 d). **A** Total protein concentration in BALF. **B** TGF-β1 concentration in BALF. **C**–**E** Relative mRNA expression levels of *Ki67*, *Ctgf*, and *Cyr61* in the left lung. **F** Representative immunofluorescence co-staining of SPC (red, AT2 cell marker) and Ki67 (green) in lung sections. White arrows indicate Ki67^+^SPC^+^ cells. **G** Quantification of the percentage of Ki67^+^SPC^+^ cells among total SPC^+^ cells. **H** Representative immunofluorescence co-staining of SPC (green) and YAP1 (red). White arrows indicate YAP1^+^SPC^+^ cells. **I** Quantification of SPC^+^ cells with nuclear YAP1. **J** Representative immunofluorescence co-staining of SPC (green) and CLDN4 (red) in lung sections. White arrows indicate CLDN4^+^SPC^+^ cells. **K** Quantification of the percentage of CLDN4^+^SPC^+^ cells among total SPC^+^ cells. **L** Representative immunofluorescence co-staining of SPC (green) and AQP5 (red). White arrows indicate AQP5^+^SPC^+^ cells. **M** Quantification of the percentage of AQP5^+^SPC^+^ cells among total SPC^+^ cells. Relative mRNA expression of *Pink1* (**N**), *Ki67* (**P**), *Ctgf* (**R**), and *Cyr61* (**S**), in the left lung, along with total protein (**O**), and TGF-β1 concentrations (**Q**), in the BALF. **T** Representative Western blotting of YAP1 in nuclear and cytoplasmic fractions. **U** Quantification of relative nuclear YAP1 protein expression. Data are presented as mean ± SD. One-way ANOVA was used for statistical analysis. Comparisons were made as follows: vs. WT+sham group, *p < 0.05, **p < 0.01, ***p < 0.001, ****p < 0.0001, ns not significant; vs. WT + I/R group, #*p* < 0.05, ##*p* < 0.01, ###*p* < 0.001, ####*p* < 0.0001; vs. NC + I/R group, $$*p* < 0.01, $$$*p* < 0.001, *p* < 0.0001, ns not significant. (Sham: sham-operated group; I/R: ischemia for 1 hour followed by 3 d reperfusion, WT wild-type group, NC airway injection of AAV-Negative Control, oePINK1 airway injection of AAV-PINK1-cDNA, shPINK1: airway injection of AAV-PINK1shRNA).

To elucidate the role of PINK1 in alveolar regeneration following LIRI, we assessed PINK1 protein levels on day 3 post-I/R by Western blotting. Compared with the sham group, PINK1 expression was significantly upregulated in the I 1 h/R 3 d group, indicating its potential involvement in lung repair after LIRI (Fig. S1F). Furthermore, IF co-staining analysis revealed significant colocalization of SPC and PINK1 following I/R, indicating that PINK1 is upregulated in AT2 cells after LIRI (Figure S1G). We then compared lung injury and repair in WT and *Pink1*^*-/-*^ mice at 3 days after I/R. Although HE staining showed very slight histological alterations (Fig. S1H), alveolar protein leakage was elevated at 3 days post-I/R (Fig. 4A). This leak was substantially mitigated in *Pink1*^*-/-*^ mice when compared with WT controls (Fig. 4A). Consistently, the I 1 h/R 3d-induced TGF-β1 levels increased in BALF (Fig. 4B), which was reversed by PINK1 deficiency, suggesting that the loss of PINK1 facilitates post-LIRI tissue repair. In contrast, no difference in IL-10 levels was detected (Fig. S1I) at day 3 post-I/R, likely because IL-10 elevation occurs at earlier inflammatory stages rather than during the repair phase. Moreover, the transcriptional levels of *Ki67*, *Ctgf*, and *Cyr61* were reduced after I/R, while PINK1 knockout markedly increased the expression of all three genes (Fig. 4C–E). These findings indicate that PINK1 deficiency enhances cellular proliferation and activates YAP signaling in the lung at reperfusion for 3 days; PINK1 may regulate post-LIRI alveolar regeneration through modulation of YAP activity.

Through IF co-staining of SPC with either Ki67 or YAP1, we further examined the effect of PINK1 on alveolar regeneration and the Hippo-YAP signaling pathway. The results showed that after I 1 h/R 3 d treatment, the proportion of SPC and Ki67-double positive cells among all SPC^+^ cells was significantly higher in *Pink1*^*-/-*^ mice than in WT mice, indicating a marked increase in proliferating AT2 cells following PINK1 knockout (Fig. 4F, G). Similarly, the proportion of AT2 cells (indicated by SPC) with YAP nuclear translocation was also significantly higher in *Pink1*^*-/-*^ mice compared to WT mice (Fig. 4H, I). We also tested the differentiation of AT2 cells by IF co-staining of SPC with either CLDN4 or AQP5. The results showed that the proportion of AT2 cells expressing CLDN4 and AQP5 was both higher in *Pink1*^*-/-*^ mice compared to WT mice after I/R treatment (Fig. 4J–M). Together, these results suggest that PINK1 knockout promotes lung tissue regeneration and repair after LIRI in mice, particularly of the alveolar epithelium.

We then exploited the tropism of adeno-associated virus type 6 (AAV6) to selectively target AT2 cells [43]. Intratracheal administration of AAV6-PINK1shRNA or AAV-PINK1-cDNA achieved AT2-specific PINK1 silencing and overexpression, respectively (Fig. S2A). The efficiency of PINK1 knockdown and overexpression in lung tissues was verified by RT-qPCR and Western blotting (Fig. 4N, T), while successful infection of AT2 cells was confirmed by immunofluorescence (Fig. S2B). HE staining showed that PINK1 knockdown enhanced, while PINK1 overexpression attenuated I 1 h/R 3d-induced lung injury (Figure S2C).

We found that PINK1 knockdown decreased LIRI-induced alveolar protein leakage, further increased the LIRI-induced TGF-β1 level increment in BALF, and increased the LIRI-induced transcriptional levels of *Ki67*, *Ctgf*, and *Cyr61* in lung tissues (Fig. 4O–S). These findings were consistent with those observed in PINK1 knockout mice. Conversely, PINK1 overexpression exacerbated alveolar protein leakage at day 3 after I/R (Fig. 4O) and downregulated the LIRI-induced increase of the proliferation- and regeneration-related marker Ki67 in lung tissue and TGF-β1 in BALF (Fig. 4P, Q). In addition, PINK1 overexpression suppressed the LIRI-induced increasing of the transcription of YAP downstream targets *Ctgf* and *Cyr61* (Fig. 4R, S). The above results further indicated that PINK1 knockdown promotes alveolar epithelial regeneration after LIRI in mice.

We determined YAP protein levels in nuclear and cytoplasmic fractions of lung tissue samples. Lung I/R induced an increase in nuclear YAP protein level, which was further enhanced by PINK1 knockdown but suppressed by PINK1 overexpression (Fig. 4T, U). These findings suggested that PINK1 knockdown further increased lung IRI-induced YAP nuclear translocation, indicating more pronounced YAP signaling activation.

Collectively, these results demonstrate that PINK1 deficiency and PINK1 knockdown facilitate YAP nuclear translocation, thereby activating downstream proliferation and regeneration programs and enhancing alveolar regeneration after LIRI. Considering that phosphorylated YAP can be degraded by ubiquitination, while non-phosphorylated YAP can enter the nucleus and participate in gene transcription regulation, the above results indicate that PINK1 may regulate the stability of cytoplasmic YAP through catalyzing the phosphorylation of YAP.

PINK1 is a critical initiator of mitophagy; consequently, PINK1 deficiency is expected to impair this process. To assess mitophagy flux, we examined key regulatory proteins at day 3 post-I/R. While p-Parkin exhibited a modest upregulation, we observed negligible changes in the ratio of LC3-II/I, as well as in the levels of p62 and mitochondrial markers (TIM23 and TOM20) (Fig. S1J, S1K). These data suggest that although I 1 h/R 3 d triggers PINK1 activation, it leads to a blockade of autophagic flux rather than complete mitophagy execution.

Notably, PINK1 deficiency attenuated the I/R-induced increase in p-Parkin but did not alter the accumulation of mitochondrial proteins, p62, or the LC3-II/I ratio (Fig. S1J, K). This indicates that while PINK1 loss diminishes PINK1 signaling, it does not rescue the I/R-induced autophagic blockage. Collectively, considering the potent activation of the YAP signaling pathway by PINK1 deficiency, we propose that during LIRI, PINK1 activation primarily facilitates YAP phosphorylation and its associated biological cascades, rather than merely serving as a driver of mitophagy.

### PINK1 knockdown promotes alveolar regeneration after LIRI via YAP

**Fig. 5:**
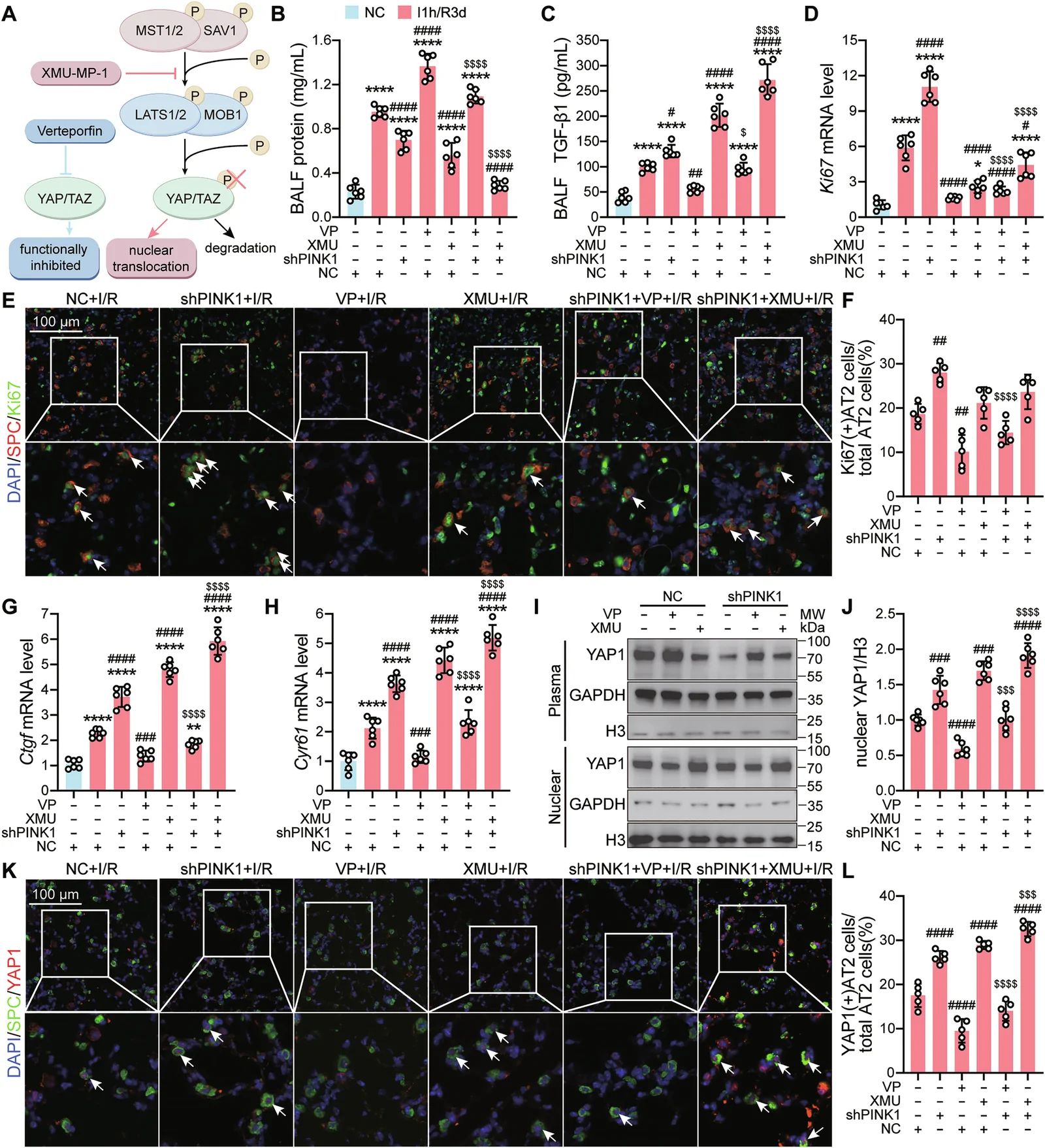
PINK1 suppresses alveolar regeneration after lung ischemia-reperfusion injury via YAP. **A** Schematic of the molecular targets of VP, an antagonist of YAP, and XMU, promoting the nuclear translocation of YAP by inhibiting the phosphorylation of LATS1/2 and MOB1. **B**–**D** BALF total protein concentration, BALF TGF-β1 concentration, and relative mRNA expression of *Ki67* in the left lung. (**E**) Representative immunofluorescence co-staining of SPC (green, AT2 cell marker) and CLDN4 (red) in lung sections. White arrows indicate CLDN4^+^SPC^+^ cells. **F** Quantification of the percentage of SPC⁺CLDN4⁺ cells among total SPC⁺ cells. **G**, **H** Relative mRNA expression of *Ctgf*, and *Cyr61* in the left lung. **I** Representative Western blotting of nuclear and cytoplasmic YAP1 in lung homogenates. **J** Quantification of nuclear YAP1 protein expression. **K** Representative immunofluorescence co-staining of SPC (green) and AQP5 (red). White arrows indicate AQP5^+^SPC^+^ cells. **L** Quantification of the percentage of SPC⁺AQP5⁺ cells among total SPC⁺ cells. Data are presented as mean ± SD. One-way ANOVA was used for statistical analysis. Comparisons were made as follows: vs. NC group, ****p < 0.0001; vs. NC + I/R group, #*p* < 0.05, ##*p* < 0.01, ###*p* < 0.001, ####*p* < 0.0001; vs. shPINK1+I/R group, $*p* < 0.05, $$*p* < 0.01, $$$*p* < 0.001, *p* < 0.0001. (VP Verteporfin, XMU XMU-MP-1, i.t. intratracheal injection, i.p. intraperitoneal injection, NC AAV-Negative Control group, shPINK1 AAV-PINK1shRNA group, I/R ischemia for 1 hour followed by 3 d reperfusion).

Next, we used the YAP agonist XMU-MP-1 (XMU) [44] and the YAP inhibitor Verteporfin (VP) [45] to modulate YAP activity. The core event of the Hippo-YAP signaling pathway is the sequential phosphorylation of YAP through a kinase cascade. Phosphorylated YAP (pYAP) is unable to translocate into the nucleus to exert its pro-proliferative function [46]. XMU reduces YAP phosphorylation by inhibiting upstream kinase activity, thereby increasing nuclear YAP accumulation and enhancing YAP-dependent transcriptional activity. In contrast, VP acts by directly inhibiting the transcriptional function of nuclear YAP (Fig. 5A). Mice were intraperitoneally injected with XMU (1 mg/kg) or VP (100 mg/kg) 30 minutes before lung I 1 h/R 3 d treatment, followed by one additional dose on day 1 and day 2 after I/R to either enhance or suppress YAP activity in vivo (Fig. S2D).

We measured the total protein levels in BALF from the left lung at day 3 after I/R. Compared with the PINK1 knockdown group (shPINK1+I/R), the YAP inhibitor VP (shPINK1+VP + I/R) reversed the reduction in alveolar protein leakage induced by PINK1 knockdown and exacerbated lung injury. Similar to the pathological changes reflected by HE staining (Figure S2E). Conversely, the YAP agonist XMU (shPINK1+XMU + I/R) further enhanced the protective effect of PINK1 knockdown in reducing alveolar injury (Fig. 5B). The YAP inhibitor VP also suppressed the increases in TGF-β1 and Ki67 induced by PINK1 knockdown (shPINK1+VP + I/R vs. shPINK1+I/R), restoring them to levels comparable to the control group (NC) (Fig. 5C, D). This was consistent with our immunofluorescence co-staining of SPC and Ki67 (Fig. 5E, F). This indicates that when YAP is inhibited, PINK1 knockdown can no longer promote lung tissue regeneration and repair. Intriguingly, we observed that in the shPINK1+XMU + I/R group, *Ki67* transcription levels did not increase further but instead were significantly lower than in the shPINK1+I/R group. We speculate that XMU-induced YAP activation may have initiated alveolar regeneration and repair earlier, and our detection time point may have missed the peak of this regenerative activity. In addition, VP reversed the increases in *Ctgf* and C*yr61* induced by PINK1 knockdown (shPINK1+VP + I/R vs. shPINK1+I/R), while XMU further promoted the transcription of both genes (shPINK1+XMU + I/R vs. shPINK1+I/R) (Fig. 5G, H).

Similarly, nuclear-cytoplasmic fractionation followed by Western blotting showed that PINK1-knockdown promoted the nuclear translocation of YAP in lung tissue at day 3 after I/R, which was further enhanced by the YAP agonist XMU (shPINK1+XMU + I/R vs. shPINK1+I/R) and was reduced by the YAP inhibitor VP (shPINK1+VP + I/R vs. shPINK1+I/R) (Fig. 5I, J). The IF co-staining revealed that the proportion of SPC and YAP1 double-positive cells was higher in AAV6-PINK1shRNA-infected mice compared to that in the control group after I/R treatment (shPINK1+ I/R vs. NC + I/R). XMU and VP, respectively, enhanced (shPINK1+XMU + I/R vs. shPINK1+I/R) and reduced (shPINK1+VP + I/R vs. shPINK1+I/R) the effect of PINK1 knockdown (Fig. 5K, L). These results strongly indicated that PINK1 knockdown activates YAP signaling and increases YAP nuclear translocation.

In summary, the promotion of alveolar regeneration after LIRI by PINK1 knockdown can be reversed by the YAP inhibitor VP, whereas the YAP agonist XMU exerts a synergistic effect. These results indicate that the regenerative effect of PINK1 knockdown after LIRI is mediated via YAP.

### In vitro validation of PINK1-mediated regulation of alveolar regeneration via YAP

Our in vitro cell experiments also supported the findings from the in vivo animal studies. In vitro, MLE-12 cells (a mouse alveolar epithelial cell line) were subjected to oxygen-glucose deprivation for 4 hours and recovery in standard culture conditions (OGD 4 h/R) to simulate ischemia-reperfusion (Fig. S3A). To determine the peak regeneration period after OGD/R treatment, we detected cell viability at different recovery time points (1 h, 6 h, 12 h, 18 h, 24 h) after OGD using CCK-8 assays. We found that MLE-12 cell viability gradually decreased within 12 h after OGD/R, with progressive cell death, followed by a gradual recovery of viability and active proliferation (Fig. S3B). Ki67 IF staining showed that the proportion of Ki67-positive cells significantly increased between 12 and 24 h after OGD/R, indicating active cell proliferation, with the highest proportion observed at 18 h after OGD/R (Figure S3C, D). Therefore, for subsequent experiments, we adopted a protocol of 4 h oxygen-glucose deprivation followed by 18 h of recovery to model post-I/R regeneration in alveolar epithelial cells.

**Fig. 6:**
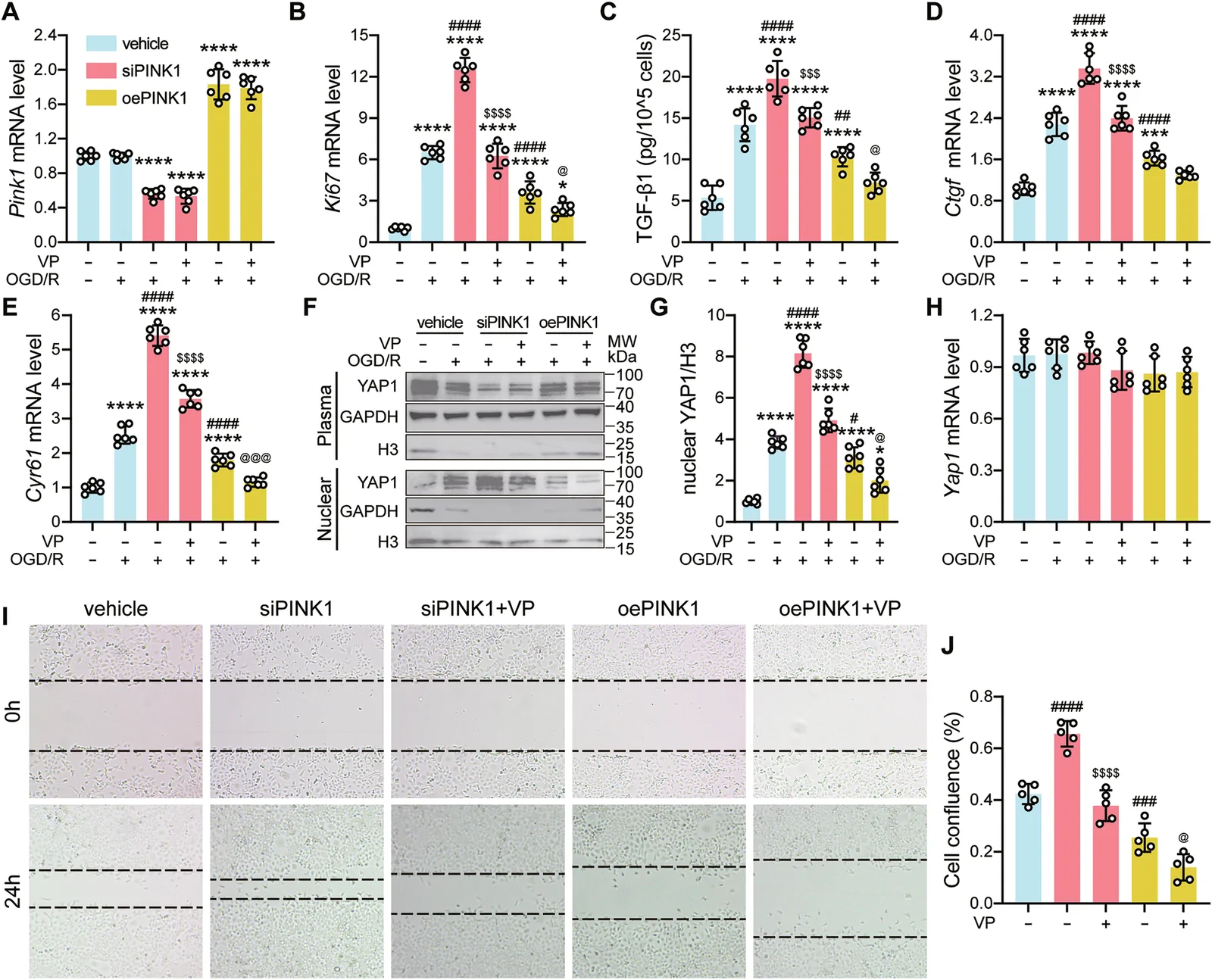
PINK1 regulates alveolar epithelial cell regeneration after ischemia-reperfusion via YAP in vitro. MLE-12 cells were subjected to oxygen-glucose deprivation for 4 h followed by 18 h recovery to standard culture with complete medium under normoxia (OGD 4 h/R 18 h). **A**–**E** Relative mRNA expression of *Pink1*, *Ki67*, *Ctgf*, and *Cyr61*, and TGF-β1 concentration in the supernatant of MLE-12 cells from each group. **F** Representative Western blotting of nuclear and cytoplasmic YAP1 in MLE-12 cells. **G** Quantification of nuclear YAP1 protein levels. **H** Relative *Yap1* mRNA expression. **I** Representative images of wound healing assay at 0 h and 24 h post-scratching in MLE-12 cells from each group. **J** Quantification of wound closure rates at 24 h. Data are presented as mean ± SD. One-way ANOVA was used for statistical analysis. Comparisons were made as follows: vs. vehicle group, *p < 0.05, **p < 0.01, ***p < 0.001, ****p < 0.0001; vs. vehicle+OGD/R group, #*p* < 0.05, ##*p* < 0.01, ###*p* < 0.001, ####*p* < 0.0001; vs. siPINK1+OGD/R group, $*p* < 0.05, $$*p* < 0.01, $$$*p* < 0.001, *p* < 0.0001; vs. oePINK1+OGD/R group, @*p* < 0.05. (vehicle: empty plasmid control group; siPINK1: PINK1 knockdown group; oePINK1: PINK1 overexpression group; VP: Verteporfin; OGD/R: OGD 4 h/R18h).

We transfected MLE-12 with PINK1siRNA to knock down PINK1 or with a PINK1 cDNA plasmid to overexpress PINK1 (Fig. 6A). Then, cells were subjected to the OGD 4 h/R 18 h treatment. We found that OGD/R increased *Ki67* transcription levels, promoted TGF-β1 secretion, and significantly elevated the transcription of *Ctgf* and *Cyr61*. PINK1 knockdown further enhanced the OGD/R-induced increment of *Ki67* transcription, TGF-β1 secretion, and *Ctgf* and *Cyr61* transcription (siPINK1 + OGD/R vs. OGD/R), whereas PINK1 overexpression (oePINK1 + OGD/R) produced the opposite changes (Fig. 6B–E). These results indicate that PINK1 knockdown promotes alveolar epithelial cell regeneration 18 h after OGD/R.

Nuclear-cytoplasmic fractionation followed by Western blotting analysis revealed that OGD 4 h/R 18 h markedly reduced cytoplasmic YAP levels while significantly increasing nuclear YAP levels in MLE-12 cells, indicating enhanced YAP nuclear translocation (Fig. 6F, G). PINK1 knockdown further increased OGD/R-induced YAP nuclear localization (siPINK1 + OGD/R vs. OGD/R), whereas PINK1 overexpression (oePINK1 + OGD/R) showed the opposite trend (Fig. 6F, G). The YAP antagonist VP reversed the increase in YAP nuclear translocation caused by PINK1 knockdown (siPINK1 + VP + OGD/R vs. siPINK1 + OGD/R) (Fig. 6F, G), confirming that PINK1 regulates alveolar epithelial cell regeneration via YAP. PINK1 knockdown enhanced YAP nuclear translocation, thereby promoting proliferation and regeneration of mouse alveolar epithelial cells after I/R. Since neither PINK1 knockdown nor overexpression altered YAP transcription levels (Fig. 6H), the regulatory effect of PINK1 on YAP nuclear localization may occur through modulation of YAP post-translational modifications or alterations in its intracellular stability.

Finally, we performed a wound-healing assay to compare the repair capacity of alveolar epithelial cells across groups. At 24 h post-injury, PINK1 knockdown (siPINK1) markedly reduced the wound area and significantly increased cell confluence compared with the control (vehicle), whereas PINK1 overexpression (oePINK1) displayed the opposite trend. Furthermore, the YAP antagonist VP reversed PINK1 knockdown-enhanced confluence (siPINK1+VP), restoring it to the control(vehicle) level (Fig. 6I, J). The results further indicated that PINK1 regulates alveolar epithelial cell regeneration after ischemia-reperfusion through YAP.

### PINK1 regulates YAP stability by modulating its phosphorylation

As mentioned above, the pro-proliferative function of YAP is regulated by its phosphorylation. Phosphorylated YAP may face cytoplasmic retention or be ubiquitinated and subsequently recognized and degraded by the proteasome [47]. Therefore, the phosphorylation of YAP is a key determinant of its functional activity.

**Fig. 7:**
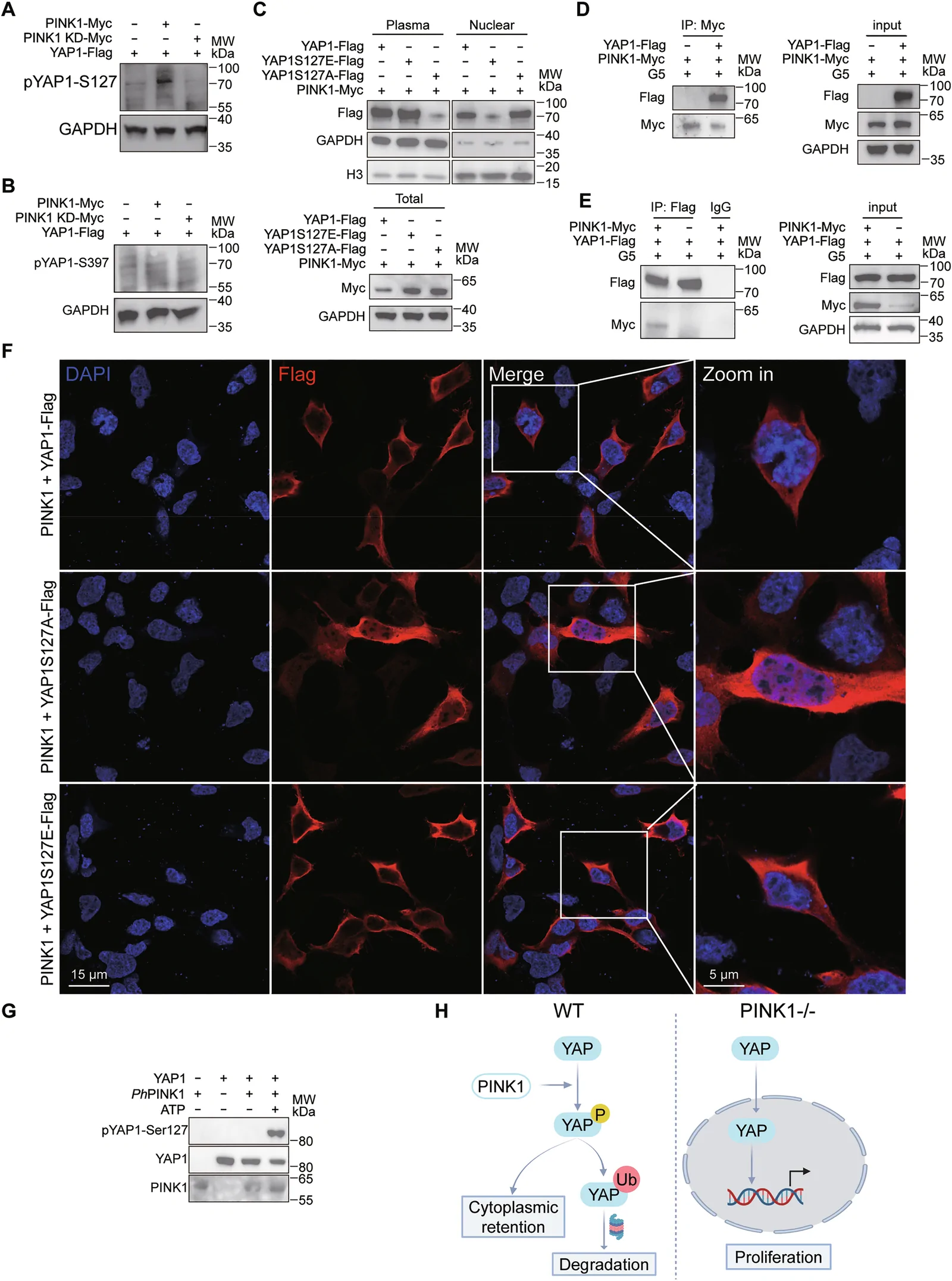
PINK1 regulates YAP stability by modulating its phosphorylation. Western blotting of pYAP1-S127 (**A**) and pYAP1-S397 (**B**) in PINK1-KO HEK293 cells transfected with Flag-tagged YAP1 and Myc-tagged PINK1 or kinase-dead PINK1 mutant (PINK1-KD). **C** Western blotting of cytoplasmic and nuclear fractions for Flag in PINK1-KO HEK293 cells transfected with Myc-tagged PINK1 and Flag-tagged wild-type YAP1 or YAP1 mutants at the Ser127 site. o-immunoprecipitation (Co-IP) assays using anti-Myc (**D**), anti-Flag (**E**), or control IgG antibodies in PINK1-KO HEK293 cells co-transfected with Myc-tagged PINK1 and Flag-tagged YAP1 and treated overnight with the deubiquitinase inhibitor G5. Corresponding Western blots for Flag, and Myc in cell lysates are shown. **F** Immunofluorescence staining of Flag in PINK1-KO HEK293 cells transfected with Flag-tagged wild-type YAP1 or YAP1 mutants at the Ser127 site, with PINK1 co-transfection. **G** Western blotting of in vitro phosphorylation assay using purified *Ph*PINK1 and YAP1 proteins. **H** Schematic model illustrating how PINK1 regulates YAP stability by promoting its phosphorylation at Ser127.

To investigate the specific mechanism by which PINK1 regulates YAP nuclear translocation, we first examined the effect of PINK1 on YAP phosphorylation. We generated a PINK1-stably knockout HEK293 cell line (PINK1^-/-^ HEK293). Then, the cells were transfected with PINK1-Myc or kinase-dead PINK1-Myc (PINK1 KD-Myc) and YAP1-Flag plasmids. We found that PINK1-expression markedly increased YAP phosphorylation at serine 127 (S127) (YAP + PINK1 vs. YAP alone), whereas co-expression of kinase-dead PINK1 (YAP + PINK1 KD) did not alter YAP S127 phosphorylation (Fig. 7A). Regardless of PINK1 expression, no significant difference was observed in YAP S397 phosphorylation (Fig. 7B). These findings indicate that PINK1 promotes YAP S127 phosphorylation in a kinase activity-dependent manner.

To confirm that PINK1, a serine/threonine kinase, directly regulates YAP stability via phosphorylation at S127, we constructed YAP-Flag plasmids with S127 site mutations to mimic phosphorylation (YAP S127E) or prevent phosphorylation (YAP S127A). Following co-transfection with PINK1-Myc in PINK1^-/-^ HEK293 cells, nuclear-cytoplasmic fractionation followed by Western blotting was performed to assess YAP nuclear localization. In the presence of PINK1, YAP S127E exhibited significantly reduced nuclear translocation compared with wild-type YAP, whereas YAP S127A showed markedly increased nuclear localization (Fig. 7C). Co-IP assays further demonstrated a direct interaction between PINK1 and YAP, as YAP-Flag was detected in Myc-tagged PINK1 immunoprecipitants, and vice versa (Fig. 7D, E). Immunofluorescence showed that when PINK1^-/-^ HEK293 cells co-expressed PINK1 with wild-type YAP, YAP had almost no nuclear localization. Conversely, co-expression of PINK1 and YAP S127A significantly increased YAP nuclear localization, while co-expression with YAP S127E resulted in minimal nuclear localization, similar to wild-type YAP (Fig. 7F). We then performed an in vitro phosphorylation assay using purified *Ph*PINK1 and YAP1 proteins. Using Western blotting, we confirmed that *Ph*PINK1 directly increases pYAP1 S127 levels in vitro (Fig. 7G). Together, these results indicate that PINK1 can directly phosphorylate YAP at S127 thereby suppressing its nuclear translocation.

In summary, our findings reveal that PINK1 regulates YAP by directly phosphorylating YAP at S127 to suppress its nuclear translocation. Loss of PINK1 increases the amount of unmodified YAP available for nuclear import, thereby enhancing cell proliferation and, at the tissue level, promoting alveolar regeneration after LIRI (Fig. 7H).

### PINK1 knockout thickens alveolar basement membrane and impairs lung function

**Fig. 8:**
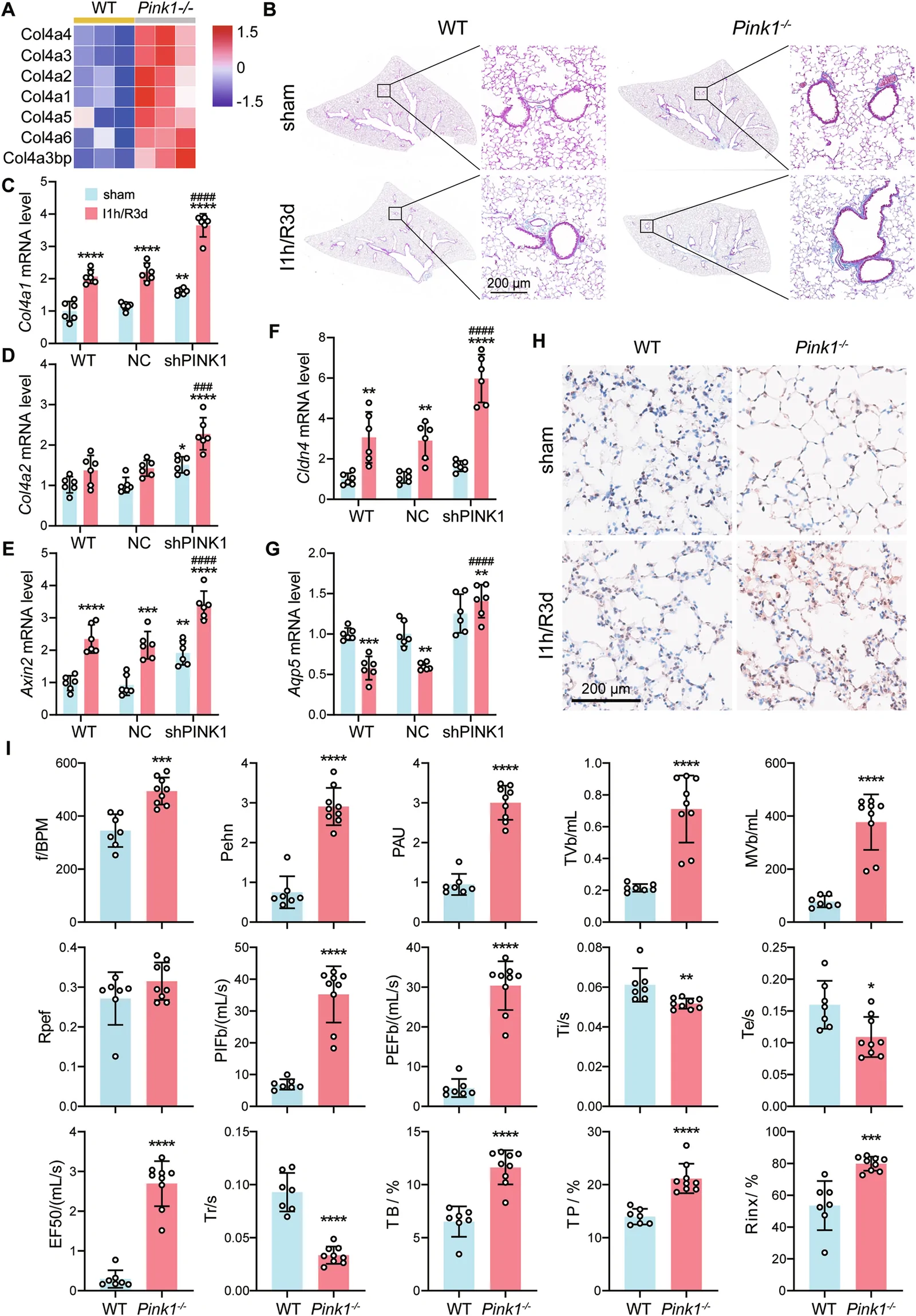
PINK1 knockout promotes alveolar basement membrane thickening and impairs lung function. **A** Heatmap of expression levels of type IV collagen-related genes in mouse lung tissue. **B** Mice were subjected to 1 h of left lung ischemia followed by reperfusion for 3 days (I 1 h/R 3 d). Masson’s trichrome staining of left lungs. **C**–**G** Relative mRNA expression of *Col4a1*, *Col4a2, Axin2, Cldn4*, and *Aqp5* in the left lung. **H** Immunohistochemical staining of COL4 deposition in lung tissue. **I** Noninvasive pulmonary function measurements in mice. Data are presented as mean ± SD. One-way ANOVA was used for statistical analysis. Comparisons were made as follows: vs. WT+sham group, *p < 0.05, **p < 0.01, ***p < 0.001, ****p < 0.0001; vs. NC + I/R group, #*p* < 0.05, ##*p* < 0.01, ###*p* < 0.001, ####*p* < 0.0001. (NC intratracheal AAV-Negative Control group, shPINK1 intratracheal AAV-PINK1shRNA group, I/R: left pulmonary hilum clamping for 1 h followed by 3 d reperfusion).

The promotion of tissue regeneration is often a double-edged sword: excessive proliferation and imperfect regeneration may impair organ function and lead to adverse outcomes such as fibrosis. Transcriptomic analysis of lung tissue from WT and *Pink1*^*-/-*^ mice revealed that *PINK1* knockout significantly upregulated the transcription of type IV collagen-related genes (Fig. 8A). Type IV collagen is a major structural component of the basement membrane in the lung and an essential element of the alveolar-capillary barrier [48]. In the lung, it is primarily secreted by endothelial and epithelial cells, particularly AT1 cells [49]. Therefore, we used AAV6 to deliver shRNA targeting PINK1 specifically to alveolar epithelial cells. Although Masson’s trichrome staining showed no significant difference between WT and *Pink1*^*-/-*^ mice (Fig. 8B), RT-qPCR showed that *Pink1* knockdown promoted lung ischemia-reperfusion (I 1 h/R 3 d)-induced upregulation of *Col4a1* and *Col4a2* expression (Fig. 8C, D). Furthermore, *Pink1* knockdown promoted I/R-induced increase in the expression of markers associated with AT2 stemness (Axin2), AT2-to-AT1 differentiation (CLDN4), and mature AT1 cell (AQP5) (Fig. 8E–G). These results suggest that *Pink1* knockdown promotes AT2-to-AT1 differentiation, which in turn may increase type IV collagen secretion and contribute to basement membrane thickening. Immunohistochemical staining confirmed that lung I/R treatment induced greater type IV collagen deposition in the basement membrane in *Pink1*^*-/-*^ mice than in WT controls (Fig. 8H). Since increased basement membrane thickness is linked to impaired lung function, we further assessed pulmonary function and observed significant functional deterioration in *Pink1*^*-/-*^ mice (Fig. 8I). Together, the above data indicated that although *Pink1* deficiency may enhance regenerative processes such as AT2-to-AT1 differentiation during the early repair phase after LIRI, sustained loss of PINK1 can result in pathological basement membrane remodeling and a long-term decline in lung function. These results indicated that PINK1 deficiency induces imperfect repair and regeneration, which may cause abnormal structural remodeling of the lung.

## Discussion

In this study, we characterized the temporal dynamics of alveolar regeneration and repair following lung ischemia-reperfusion injury (LIRI) in mice. Our findings revealed that *Pink1* deficiency significantly enhanced alveolar regeneration during the early repair phase post-LIRI, primarily by promoting nuclear accumulation of YAP and thereby augmenting regenerative signaling. Mechanistically, we demonstrated that PINK1 directly phosphorylates YAP at Ser127, leading to its cytoplasmic retention. However, despite the accelerated early regeneration observed in *Pink1*-deficient mice, the repair process remained incomplete and ultimately resulted in aberrant basement membrane remodeling.

First of all, a foundational finding is that we refined the temporal course of alveolar epithelial injury and repair after LIRI in mice. Although various animal models have been established to investigate lung regeneration following injury, differences in injury severity and underlying mechanisms necessitate a precise characterization of the temporal dynamics of damage and repair for each specific model. For instance, in lipopolysaccharide (LPS)-induced acute lung injury, the injury phase spans 0–48 hours, followed by the initiation of the repair phase at 48 hours [50]; whereas in bleomycin-induced lung injury, the acute injury phase extends from 0 to 7 days before repair begins [51]. We employed a murine left pulmonary hilum clamping model and an oxygen-glucose deprivation/recovery (OGD/R) model in MLE-12 cells to simulate the process of LIRI. We demonstrated that regenerative activity peaked at day 3 in the murine I/R model and 18 hours after culture condition recovery in the OGD/R cell model. These findings refine our current understanding of the temporal course of alveolar epithelial injury and repair following LIRI, and offer critical reference points for identifying optimal time windows to evaluate alveolar regeneration.

Secondly, we found that *Pink1* deficiency promoted alveolar regeneration after LIRI. Previous studies on the role of PINK1 in lung injury have primarily focused on the mechanisms of injury initiation and progression, with particular emphasis on PINK1/Parkin-mediated mitophagy [52–54]. In this study, we demonstrate for the first time that PINK1 also regulates alveolar epithelial regeneration following LIRI by increasing nuclear YAP accumulation and enhancing regenerative signaling, a function that depends on its serine/threonine kinase activity but is independent of mitophagy.

Thirdly, we identified a novel phosphorylation substrate of PINK1 and its specific phosphorylation site. We found that PINK1 phosphorylates YAP, promoting its cytoplasmic retention. PINK1, as a multi-substrate serine/threonine kinase, has been shown to exert its kinase activity in the cytoplasm and phosphorylate a variety of protein substrates, including Parkin, Bcl-xL, HIF-3α, and tumor protein p53 [55]. We revealed that PINK1 can directly phosphorylate YAP at Ser127. Phosphorylation at this residue is known to enable recognition and binding by 14-3-3 proteins, resulting in the cytoplasmic retention of YAP [47]. In contrast, PINK1 showed almost no effect on YAP phosphorylation at Ser397, suggesting it does not mediate YAP ubiquitination and proteasomal degradation, which are typically driven by this site [23].

Finally, our study focused on alveolar epithelial regeneration during the early repair phase following LIRI, demonstrating that PINK1 promotes lung regeneration at day 3 post-injury. However, it is important to note that enhanced early regeneration and repair do not necessarily translate into improved long-term outcomes [17]. Although AT2 cells are progenitors of the alveolar epithelium, in certain contexts, impaired or incomplete differentiation of AT2 to AT1 cells can lead to excessive AT2 proliferation and concomitant activation of fibrotic pathways, ultimately increasing the likelihood or severity of pulmonary fibrosis [56–58]. Moreover, YAP downstream targets such as CTGF stimulate fibroblast proliferation and collagen deposition, while CYR61 exerts similar functions [59]. Their sustained or excessive activation can drive fibrosis and adverse remodeling [60]. Consistently, our transcriptomic analysis revealed significant upregulation of collagen type IV-related genes in the lungs of *Pink1*^*-/-*^ mice compared with WT controls. Therefore, the long-term consequences of PINK1 deletion after LIRI warrant further investigation. Taken together, these findings highlight PINK1 as a context-dependent regulator of alveolar repair, acting to enhance early regeneration but carrying a potential risk of maladaptive fibrosis in the long term.

This study has several limitations. In the present work, all PINK1 interventions were administered prior to model induction. If PINK1 modulation were applied after model establishment-namely, following the onset of LIRI but before the initiation of alveolar epithelial repair- using small-molecule PINK1 inhibitors or activators, or supplementation with exogenous PINK1, such an approach could yield stronger evidence for the role of PINK1 in promoting post-injury alveolar epithelial regeneration and possess greater translational potential. Furthermore, literature indicates that following intratracheal administration, AAV6-mediated infection involves Club cells, ciliated cells, and AT2 cells [43]. Although AT2 cells are the primary target cells, and our in vitro experiments have confirmed the role of PINK1 in AT2 cells, more AT2-specific manipulation is needed to rule out the influence of other cell types in the future. Finally, the underlying mechanisms driving the abnormal basement membrane remodeling induced by PINK1 deficiency warrant further investigation.

In summary, our study delineated the temporal dynamics of alveolar epithelial injury and repair following LIRI in mice. We revealed that PINK1 directly phosphorylates YAP to regulate its intracellular localization. PINK1 deficiency increases the levels of non- phosphorylated YAP in alveolar epithelial cells, facilitating its nuclear translocation and thereby promoting alveolar regeneration in the post-LIRI setting. However, prolonged loss of PINK1 may drive maladaptive tissue remodeling, underscoring the need for balanced regulation of this pathway during LIRI repair.

## Supplementary information

Methods and materials

figure legends of figure S1, S2 and S3

Figure S1

Figure S2

Figure S3

Original western blots

## Data Availability

The data that support the findings of this study are were submitted to the China National Center for Bioinformation under the accession number PRJCA070889 for the RNA-seq. All the data are publicly available. Further inquiries can be directed to the corresponding author.
