# Supplementary material for: PINK1 deficiency enhances alveolar regeneration and basement membrane remodeling by reducing YAP phosphorylation during lung ischemia-reperfusion injury
Source: Cell Death Dis. 2026 Aug 5;17(1):929. doi: 10.1038/s41419-026-09149-3 (PMC13724675; doi:10.1038/s41419-026-09149-3)

Original western blots

Figure3

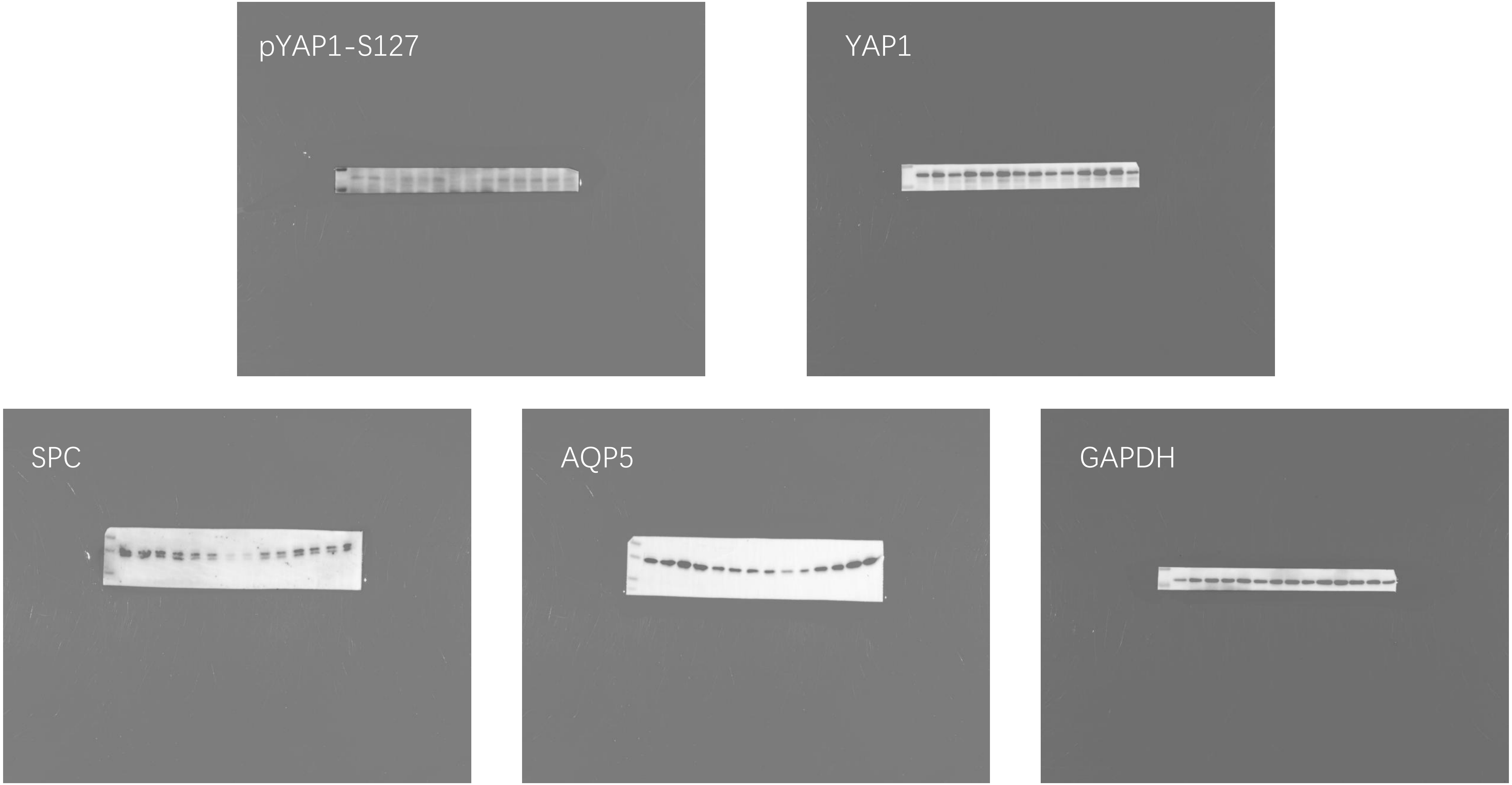

Figure4

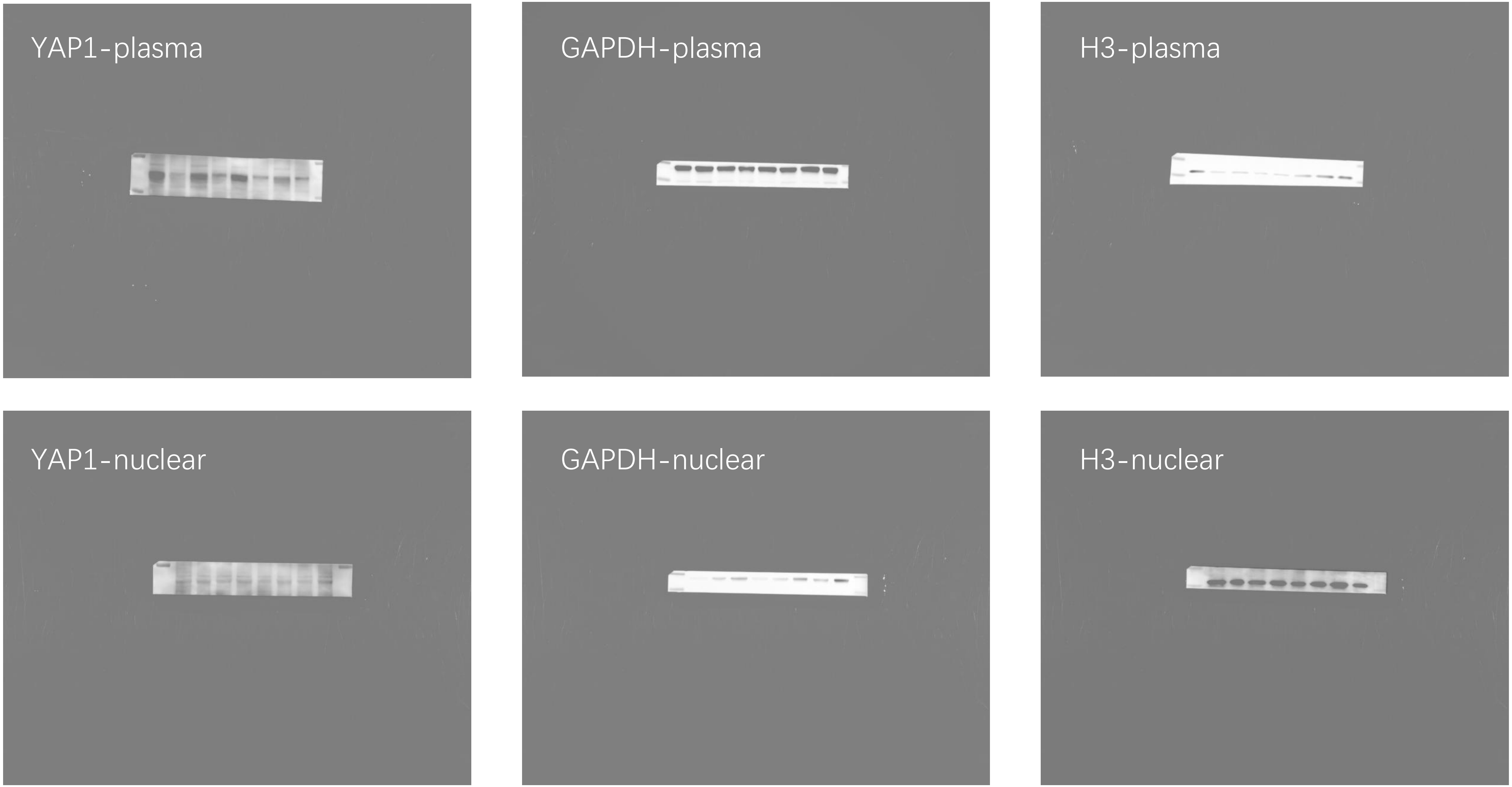
YAP1-plasma
GAPDH-plasma
H3-plasma
YAP1-nuclear
GAPDH-nuclear
H3-nuclear

Figure4

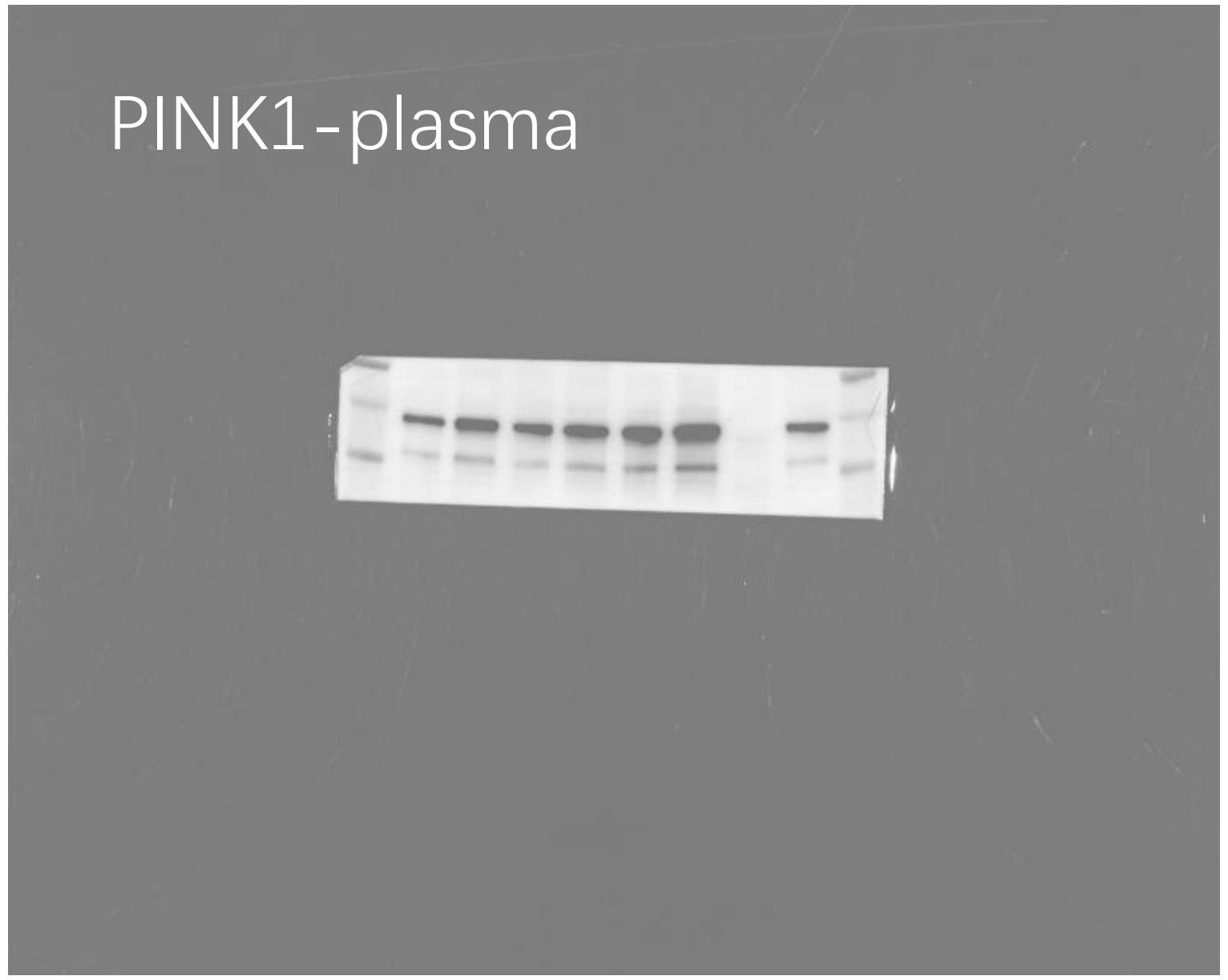

Figure5

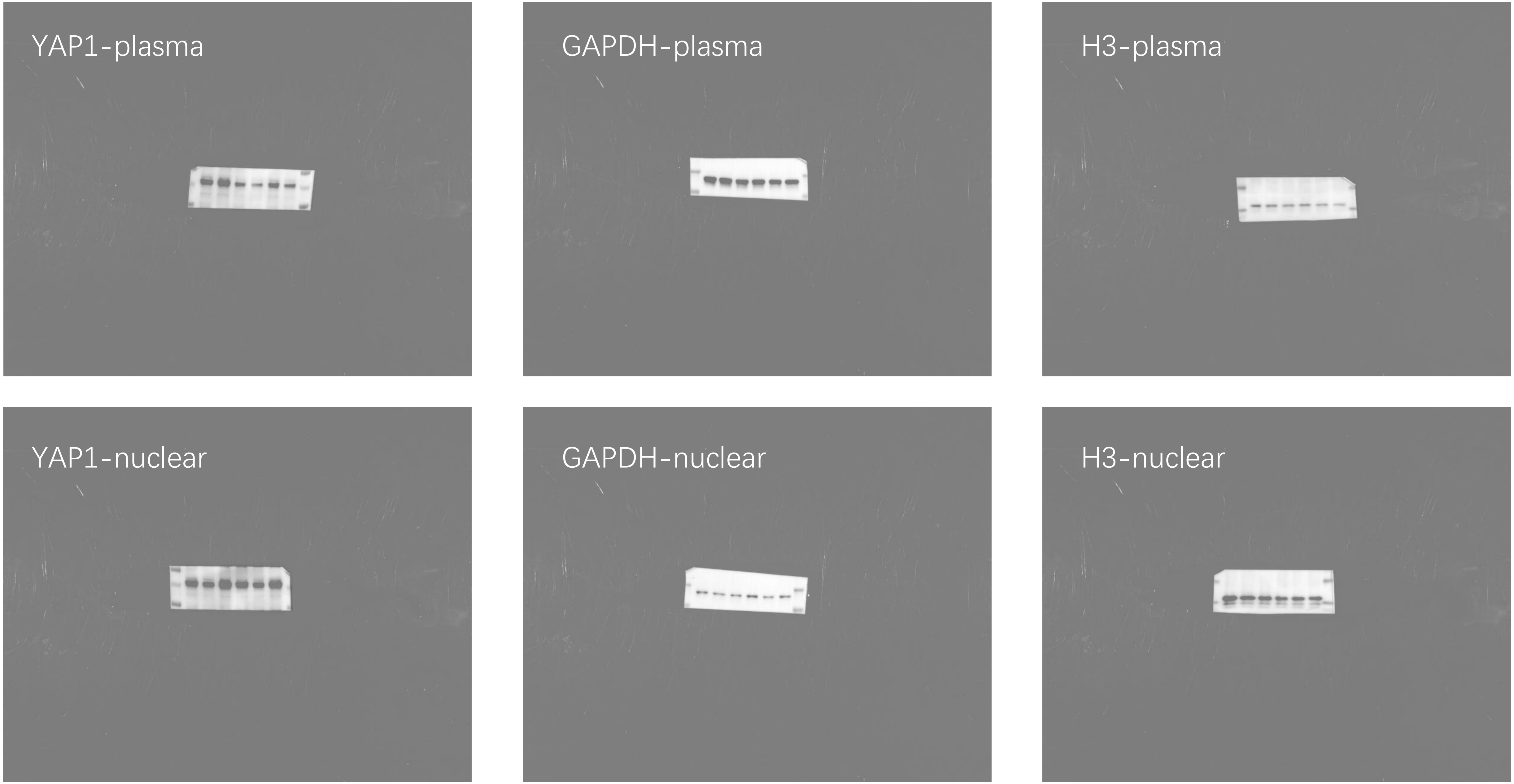
YAP1-plasma
GAPDH-plasma
H3-plasma
YAP1-nuclear
GAPDH-nuclear
H3-nuclear

Figure6

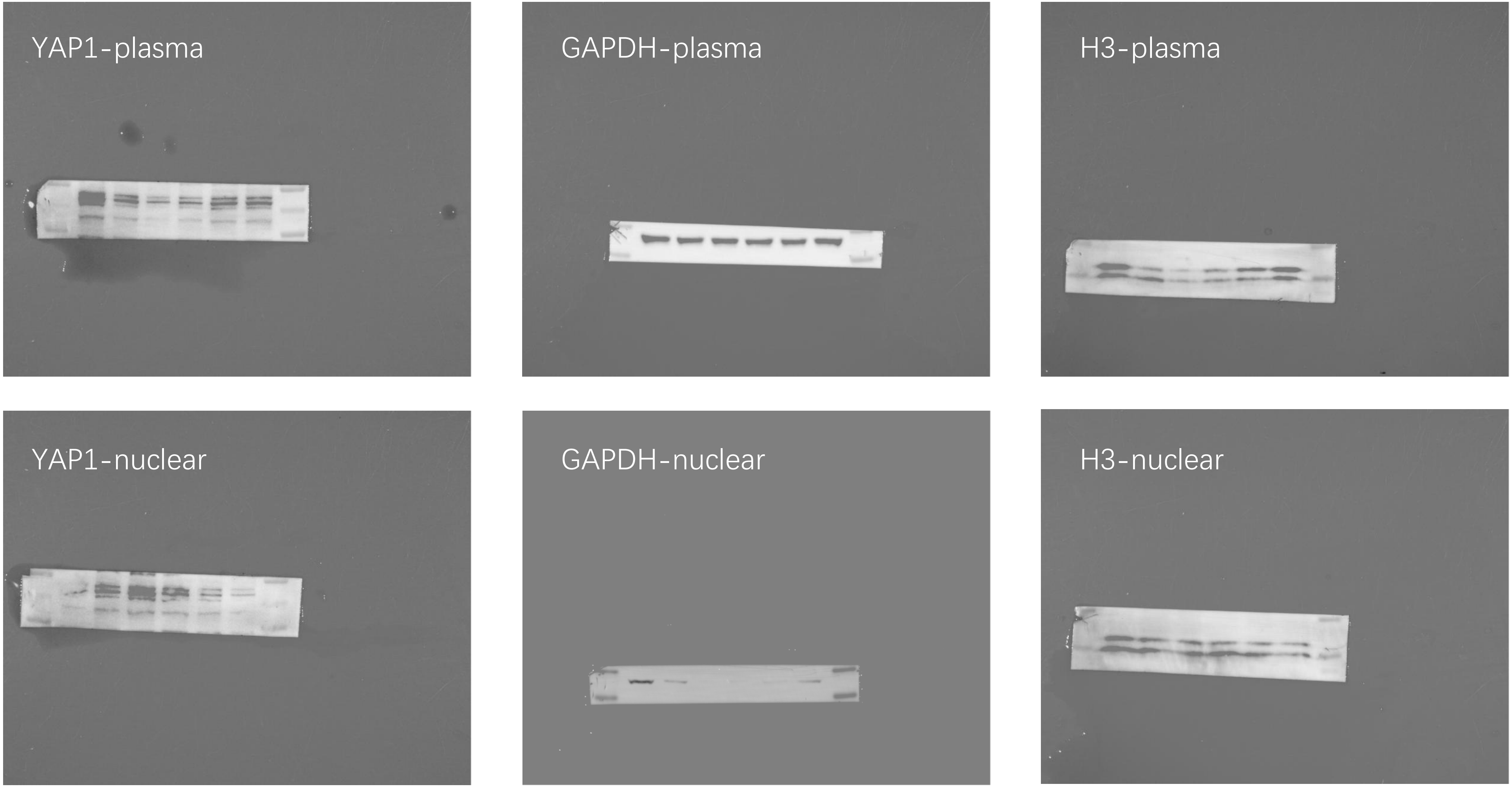
YAP1-plasma
GAPDH-plasma
H3-plasma
YAP1-nuclear
GAPDH-nuclear
H3-nuclear

Figure7A-B

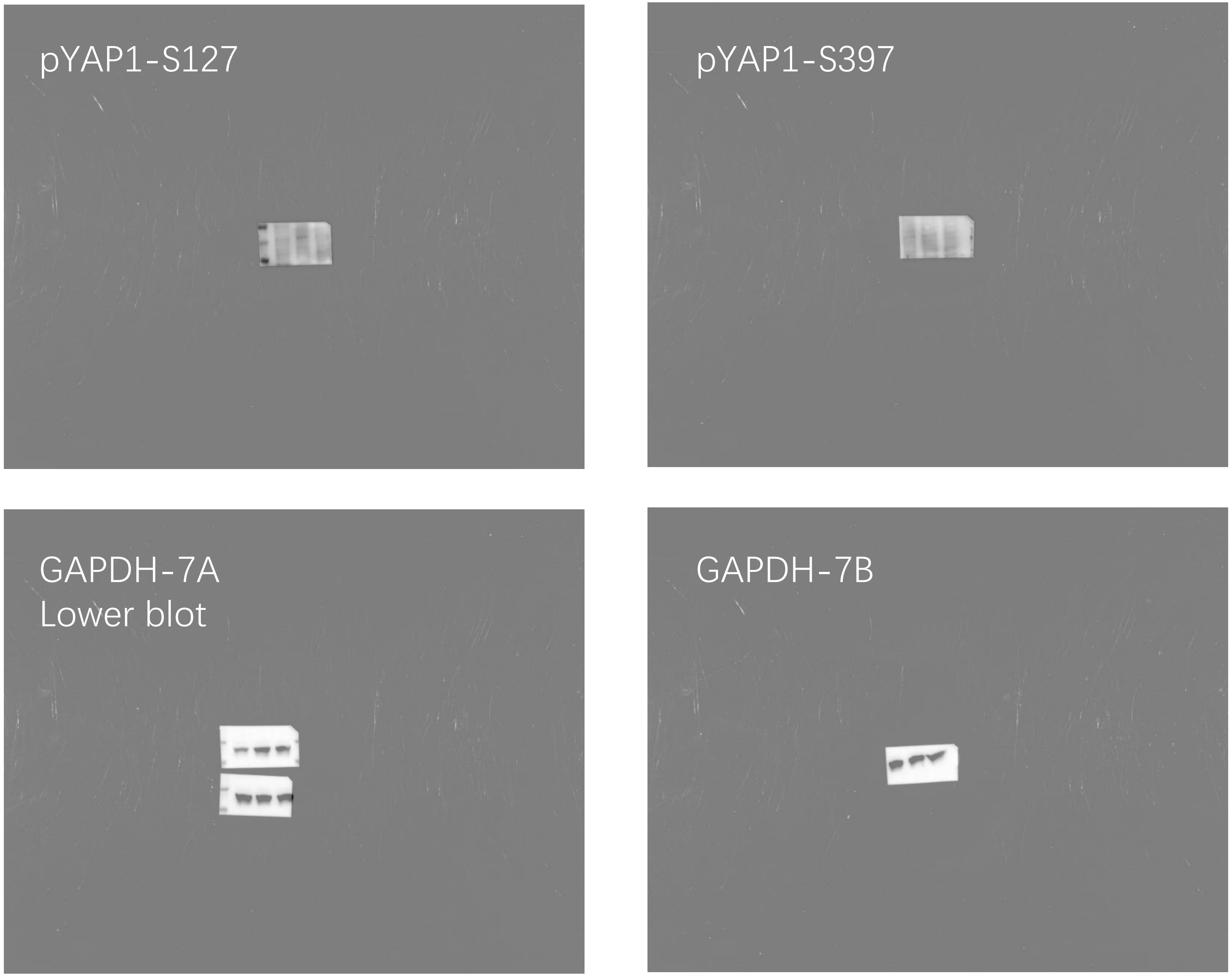

Figure7C

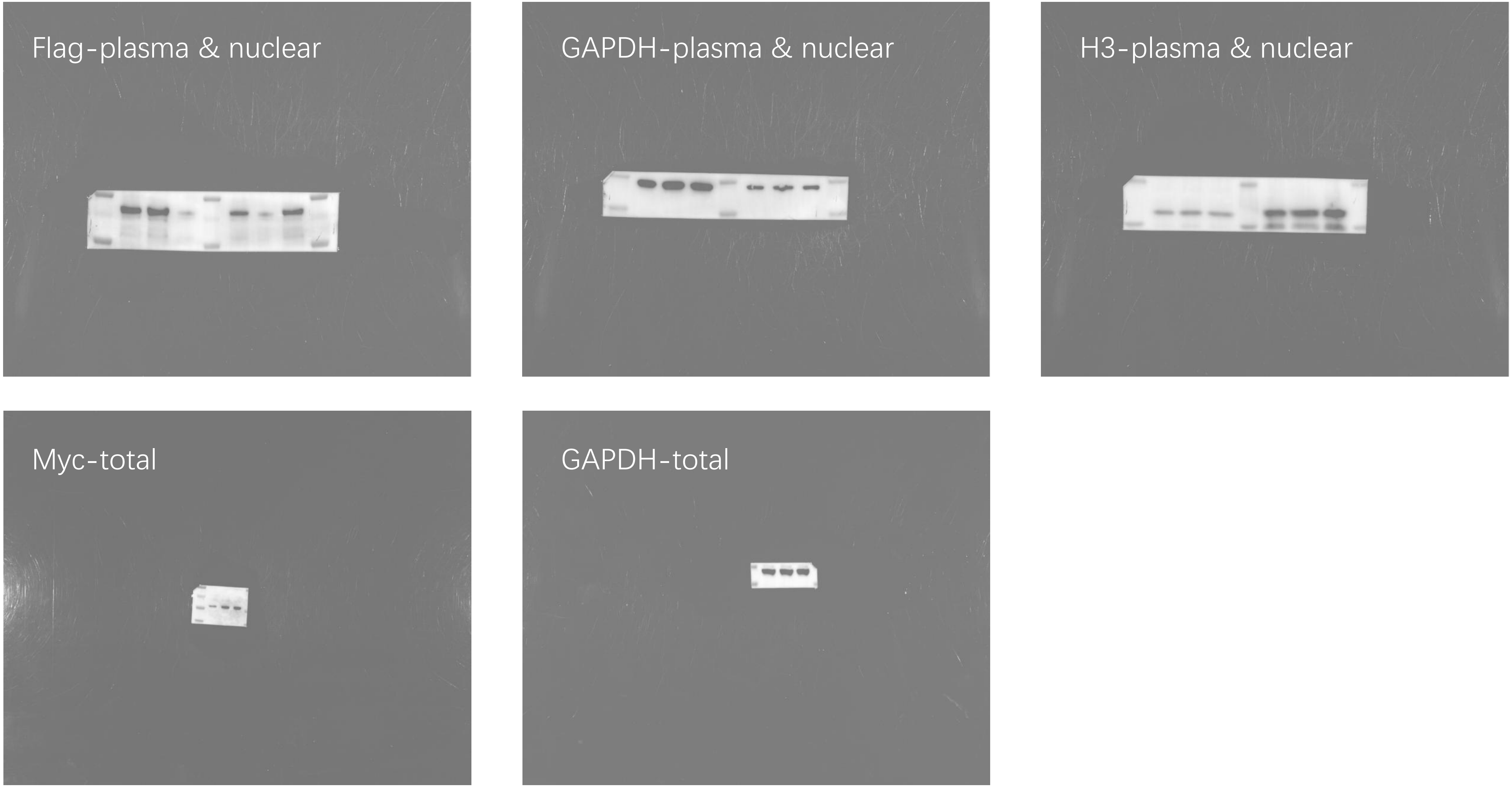
Flag-plasma & nuclear
GAPDH-plasma & nuclear
H3-plasma & nuclear
Myc-total
GAPDH-total

Figure7D

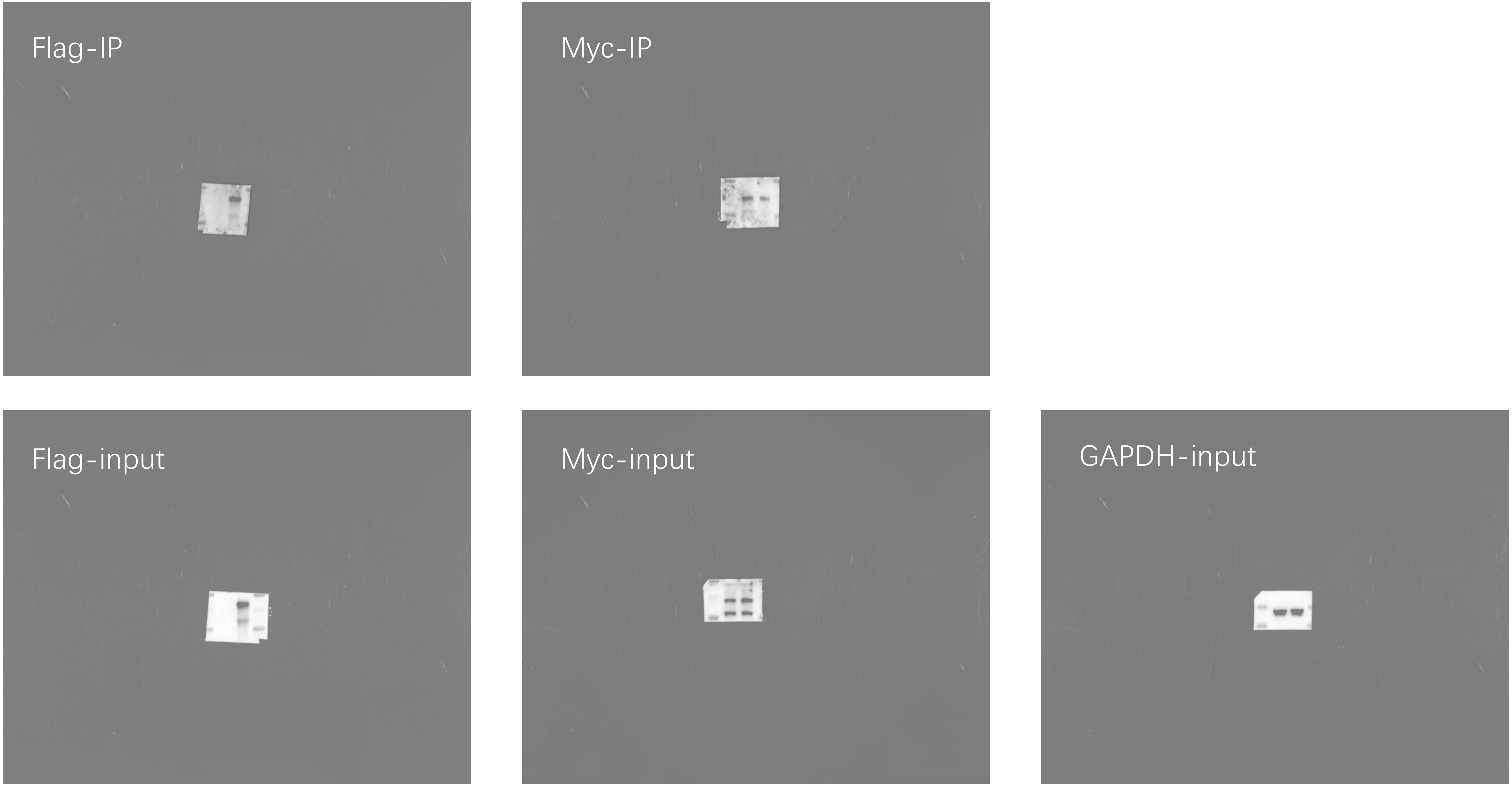

Figure7E

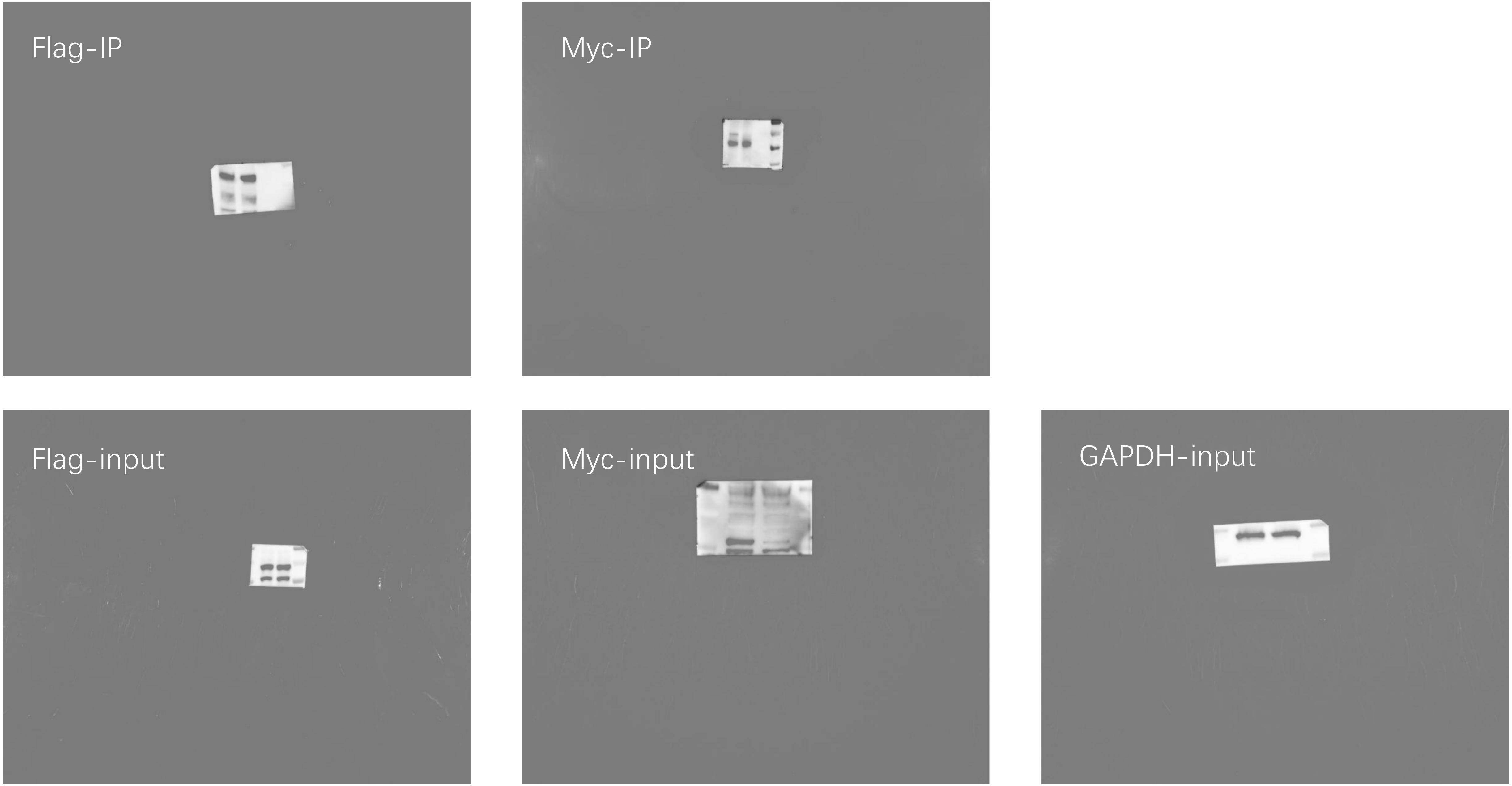

Figure7G

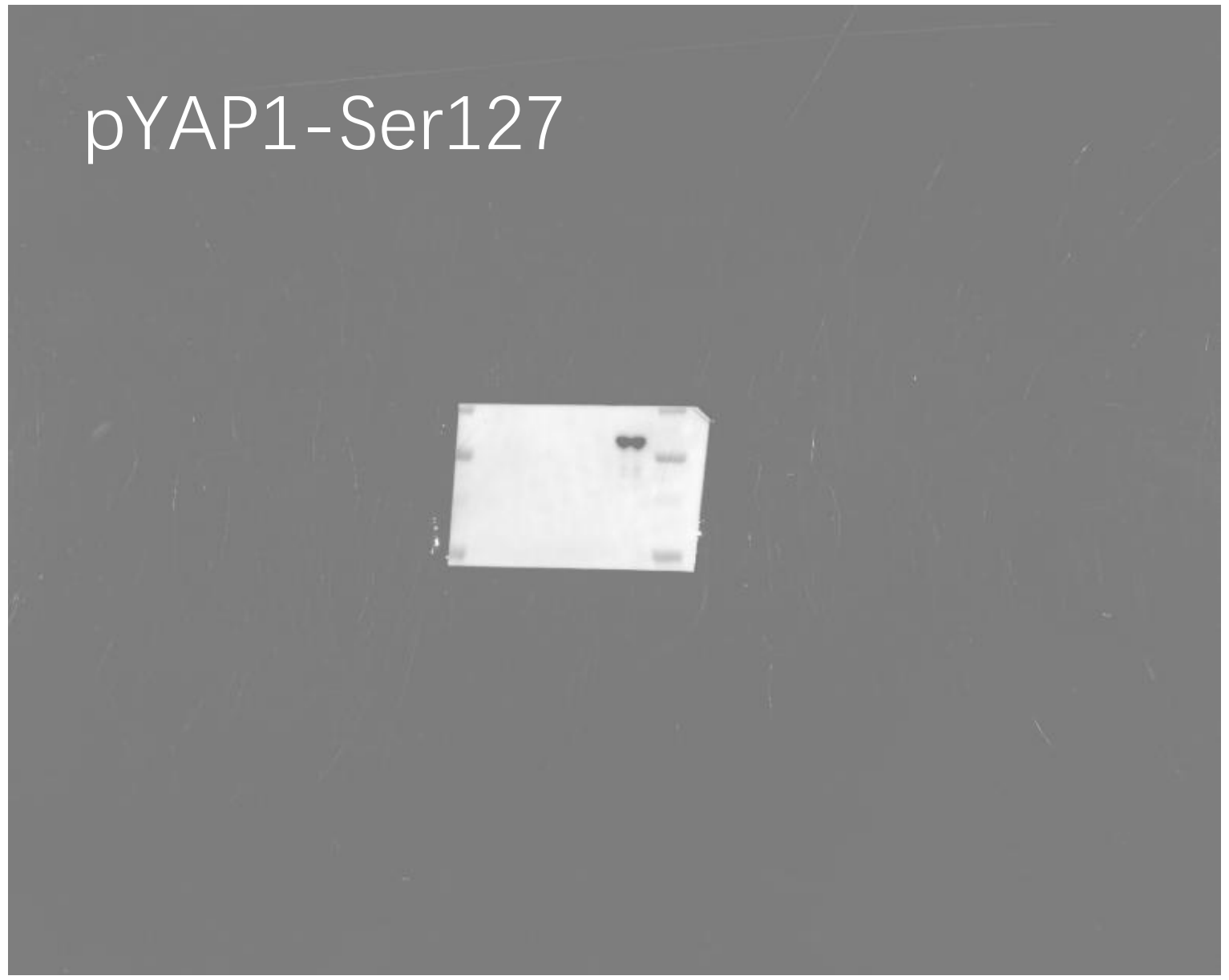

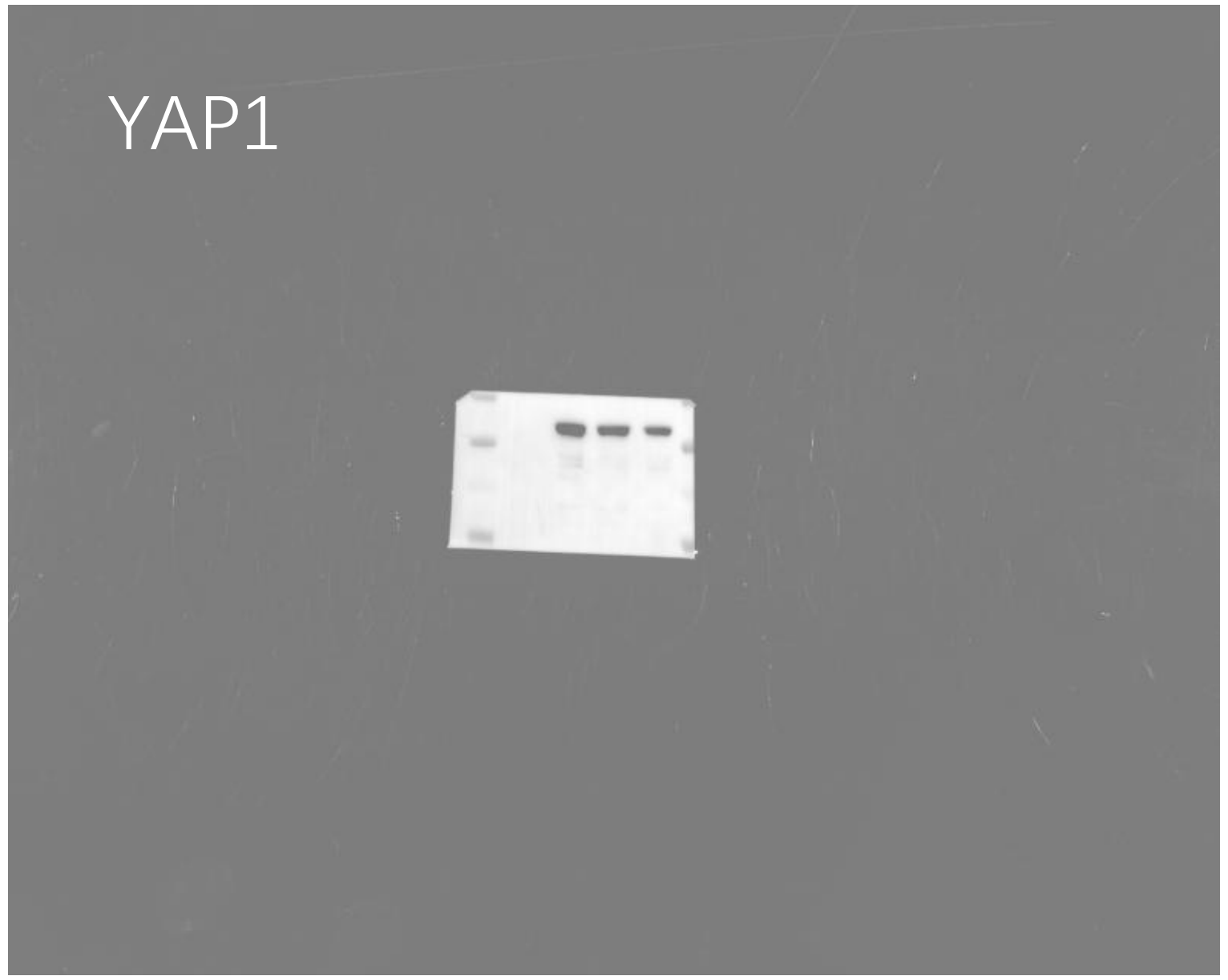

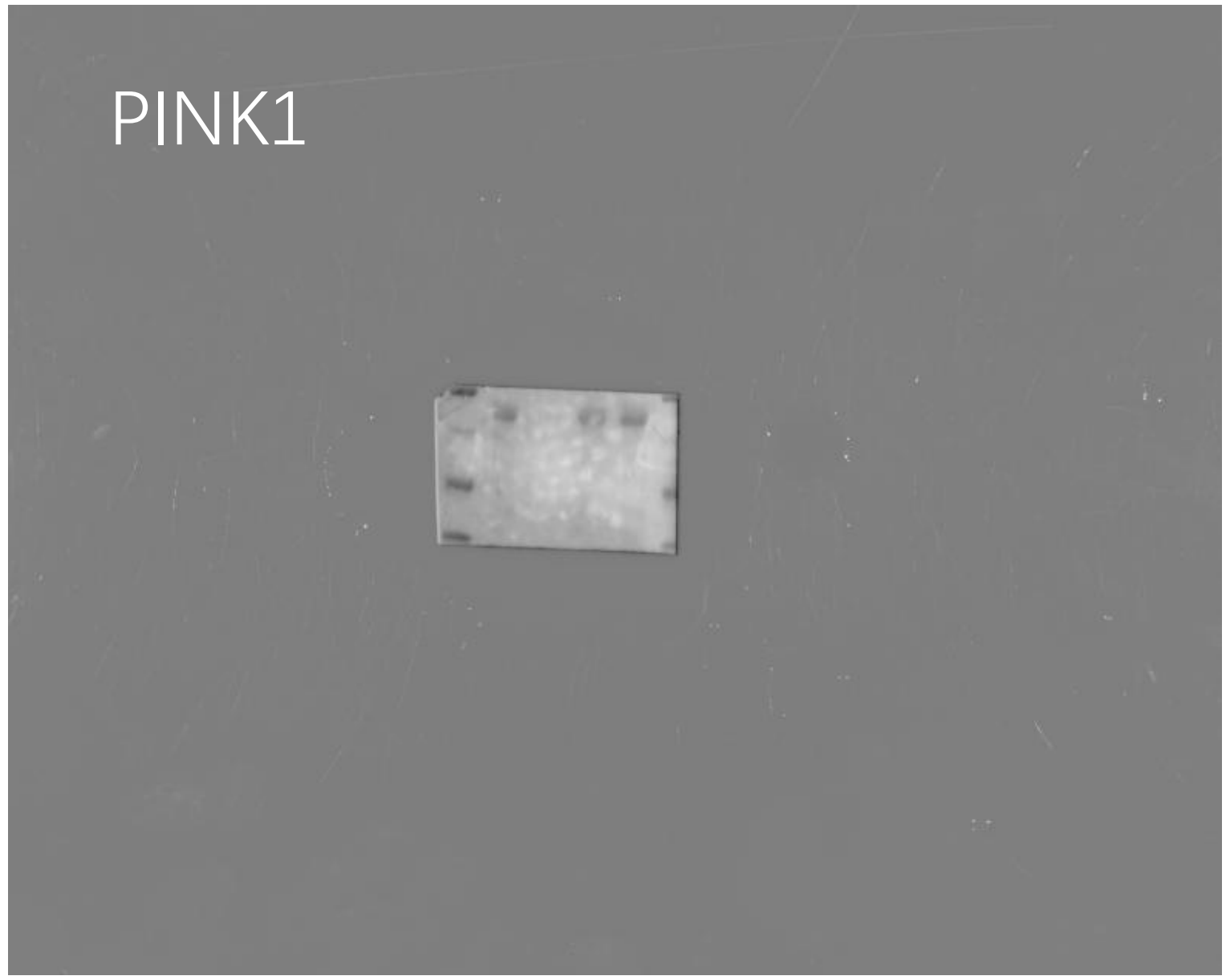

FigureS1

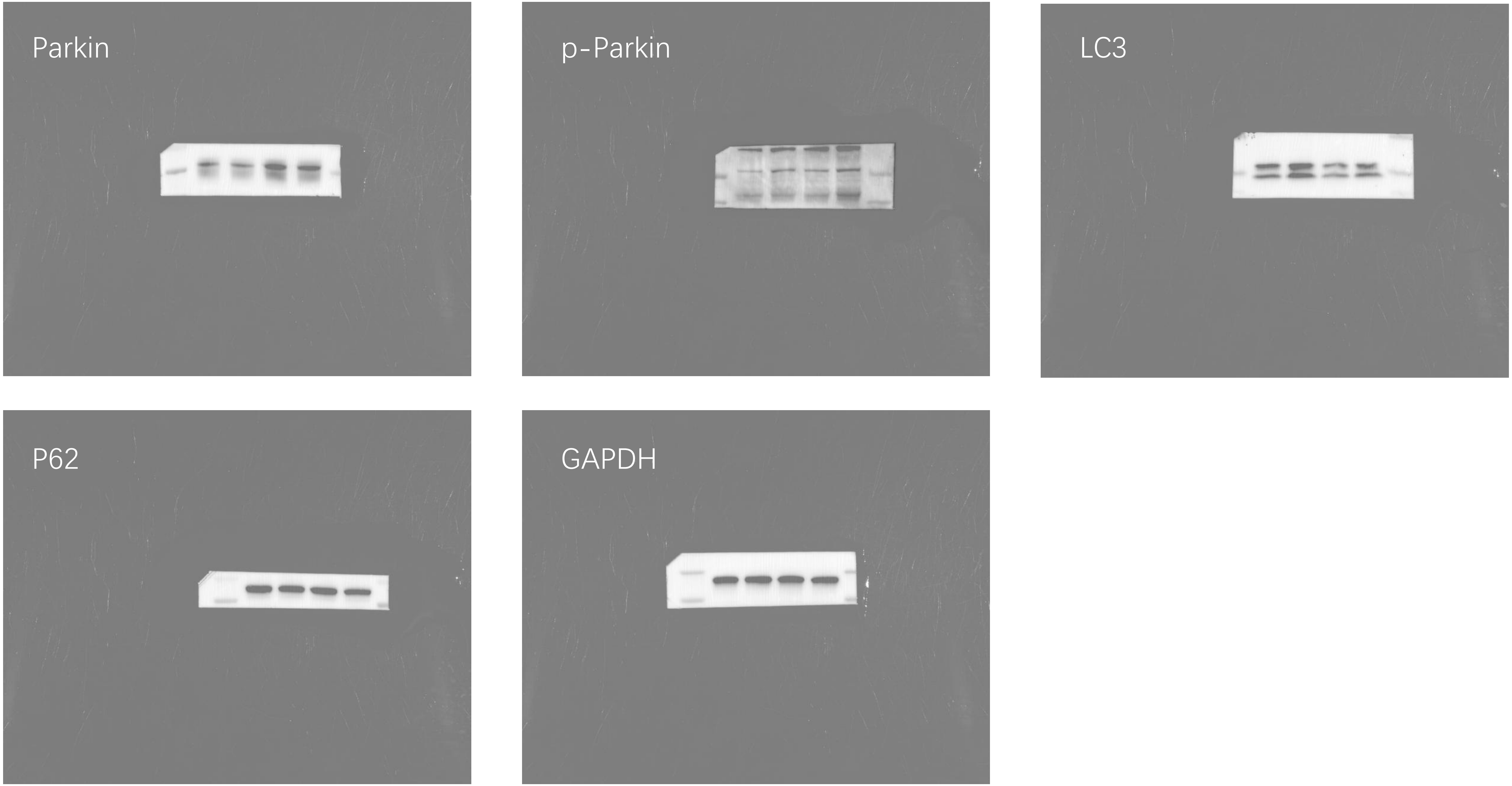

FigureS1

TOM20

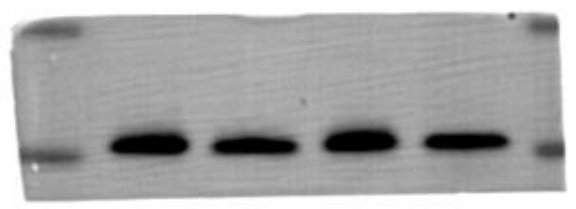

TIM23

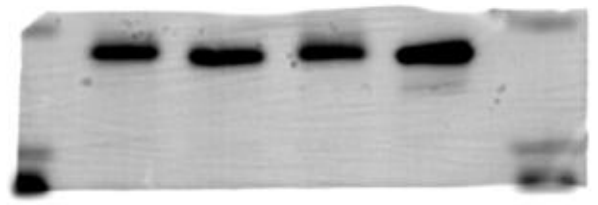

Supplement: Supplementary file 1 [file 41419_2026_9149_MOESM6_ESM.pdf]
