# Supplementary material for: PINK1 deficiency enhances alveolar regeneration and basement membrane remodeling by reducing YAP phosphorylation during lung ischemia-reperfusion injury
Source: Cell Death Dis. 2026 Aug 5;17(1):929. doi: 10.1038/s41419-026-09149-3 (PMC13724675; doi:10.1038/s41419-026-09149-3)
Supplement: Supplementary file 2 [file 41419_2026_9149_MOESM1_ESM.docx]

**Animals**

All experiments were performed in male mice. Wild-type C57BL/6J mice (8–12 weeks old) were obtained from the Laboratory Animal Center of Zhejiang Academy of Medical Sciences (Hangzhou, China). *Pink1^-/-^* mice were generated following previously established methods^1^. To specifically knock down or overexpress PINK1 in the airway, adeno-associated virus (AAV) 6 was used as vectors to delivery PINK1shRNA or full-length PINK1cDNA. A neck incision was made following anesthesia to expose the trachea, and 0.05 ml of AAV-PINK1shRNA or AAV-full-length PINK1cDNA was administered via intratracheal injection. Stable expression was achieved after 3 weeks. All animals were housed in the Laboratory Animal Center of Zhejiang University under specific pathogen-free (SPF) conditions, with free access to food and water, at 23–25 °C, 50% relative humidity, and a 12 h light/dark cycle. Experimental protocols were approved by the Institutional Animal Care and Use Committee of Zhejiang University (ZJU20240698). All procedures complied with the Guide for the Care and Use of Laboratory Animals published by the U.S. National Institutes of Health. Sample sizes were determined based on previous experience, pilot experiments, and published studies using similar models. No statistical method was used to predetermine sample size, and efforts were made to minimize animal use while ensuring sufficient statistical power to detect biologically relevant differences. Animals were randomly allocated to different groups and no blinding was done.

**Mouse model of LIRI**

Mice were anesthetized with an intraperitoneal injection of sodium pentobarbital (60 mg/kg), intubated under direct visualization, and connected to a ventilator. Animals were positioned in right lateral decubitus, the fur over the left chest was shaved, and the ribs were exposed by sequential incision. The intercostal muscles at the third intercostal space were cut, and the ribs were retracted to expose the left hilum. After transection of the left inferior pulmonary ligament, the left pulmonary hilum was carefully dissected and occluded with a microvascular clip, and the surgical field was covered with a saline-soaked cotton ball. After 1 h of ischemia, the clip was removed to initiate reperfusion, and the thoracic cavity was closed by layered suturing. Mice were returned to the prone position and mechanically ventilated until spontaneous respiration was restored, then the tracheal tube was removed. Animals were euthanized at 0 h, 6 h, 1 day, 3 days, 7 days, or 14 days after reperfusion.

**Pharmacological interventions**

Mice received an intraperitoneal injection of verteporfin (VP; 100 mg/kg, Cat#V408385, Aladdin, Shanghai, China) or XMU-MP-1 (1 mg/kg, Cat#HY-100526, MedChemExpress, USA) 30 min before left lung hilum clamping. Additional doses (VP 100 mg/kg; XMU-MP-1 1 mg/kg) were administered every 24 h after reperfusion.

**Bronchoalveolar lavage fluid (BALF) collection**

Following thoracotomy, the heart was removed. A cotton thread was passed posterior to the apex of the right lung and exteriorized at the inferior pulmonary ligament, then tied to ligate the right hilum. The trachea was incised, and a blunt syringe needle was inserted. A volume of 0.3 mL sterile saline was gently instilled into the left lung and withdrawn until resistance was felt. The BALF was collected into a 2 mL EP tube. This procedure was repeated three times. Samples were immediately snap-frozen in liquid nitrogen and stored at -80 °C until analysis.

**Histological examination**

Freshly isolated lung tissues were fixed in 4% paraformaldehyde for 24 h. The paraffin-embedded lung tissue was cut into 4-μm sections. The slices were treated with HE or Masson's trichrome staining. The inflammatory response and injury in the lungs were observed and assessed after staining.

Acute lung injury (ALI) was scored according to the following criteria as previously reported, with five randomly selected fields per section scored independently on neutrophils in alveolar space, neutrophils in interstitial space, hyaline membranes, airway debris, alveolar septal thickening. The injury score was calculated with higher scores indicating more severe injury^2^.

Pulmonary vascular edema was quantified by calculating the ratio of perivascular edema thickness to vascular diameter, with eight vessels randomly selected per sample and measured using ImageJ (NIH, USA) ^3^. Airway wall thickness was determined by measuring the height of columnar epithelial cells in tangentially sectioned small airways^4^. Briefly, eight airways were randomly selected, and the mean value of 12 epithelial cells per airway was used for statistical analysis.

**Immunofluorescence staining**

After routine deparaffinization, rehydration, and antigen retrieval, lung sections were incubated overnight at 4 °C with the following primary antibodies: rabbit anti-SPC (Cat#ab211326, Abcam, Cambridge, UK; 1:200), rat anti-Ki67 (Cat#14-5698-82, Invitrogen, USA; 1:100), mouse anti-YAP1 (Cat#A1002, ABclonal, Wuhan, China; 1:200), rabbit anti-CLDN4 (Cat#36-4800, Invitrogen, USA; 1:100), rabbit anti-AQP5 (Cat#ab78486, Abcam, Cambridge, UK; 1:200). On the following day, sections were washed with PBS and incubated with the corresponding secondary antibodies: Cy3-conjugated donkey anti-mouse (Cat#AP129), Cy3-conjugated donkey anti-rabbit (Cat#AP182C), Cy3-conjugated donkey anti-rat (Cat#AP189C), FITC-conjugated goat anti-mouse (Cat#AP124F), FITC-conjugated goat anti-rabbit (Cat#AP132F), and FITC-conjugated goat anti-rat (Cat#AP183F) (all from Millipore, MA, USA; diluted 1:200). Nuclei were counterstained with DAPI (Cat#P36935, Thermo Fisher Scientific, MA, USA). Images were acquired and processed using a confocal fluorescence microscope (FV100, Olympus, Japan).

**Immunohistochemistry**

Paraffin-embedded lung sections were deparaffinized, rehydrated, and subjected to antigen retrieval in citrate buffer (pH 6.0). Endogenous peroxidase activity was blocked with 3% hydrogen peroxide. Sections were incubated overnight at 4°C with the primary antibody: rabbit anti-Collagen IV (Cat#ab6586, Abcam, Cambridge, UK; 1:200), followed by secondary antibody: HRP-conjugated goat anti-rabbit (Cat#A0277, Beyotime Bio, Shanghai, China; 1:200). Signals were developed using DAB (Cat#ZLI-9018, ZSGB-Bio, Beijing, China). Images were acquired under a light microscope (BX51, Olympus, Japan).

**Cell culture and treatment**

MLE-12 cells (a murine alveolar epithelial cell line; Wuhan Shine, China) were cultured in DMEM/F12 medium (Cat#C11995500BT, Gibco, USA) supplemented with 10% fetal bovine serum (FBS; Cat#10091-148, Gibco, USA) and 1% penicillin-streptomycin (Cat#P1400, Solarbio, Beijing, China) at 37 °C in a humidified atmosphere containing 5% CO₂. PINK1^-/-^ HEK293 cells were prepared using CRISPR-Cas9 gene-editing technology and maintained in DMEM medium (Cat#C11995500BT, Gibco, USA) with the same supplements and culture conditions.

**Construction of the oxygen–glucose deprivation/recovery (OGD/R) model**

Cells were seeded into culture plates at the desired density per well. After cell attachment, the medium was replaced with glucose-free DMEM, and the plates were placed in a custom-made OGD/R chamber. The chamber was sealed and connected to an inlet supplying a gas mixture of 95% N₂ and 5% CO₂, with the outlet connected to a water-sealed beaker to allow gas displacement. Cells were incubated under oxygen-glucose deprivation conditions at 37 °C for 4 h. Subsequently, the glucose-free medium was replaced with complete MLE-12 culture medium, and cells were transferred to a 37 °C incubator with 95% air-5% CO₂ for recovery to standard cell culture condition for the indicated durations.

**Cell viability assay**

Cell viability was assessed using the Cell Counting Kit-8 (CCK-8; Cat#C0038, Beyotime Bio, Shanghai, China) according to the manufacturer’s instructions. Briefly, MLE-12 cells were seeded into 96-well plates at a density of 4 × 10^4^ cells per well. After OGD/R treatment, cells were incubated with CCK-8 solution for 2 h. The absorbance was measured at 450 nm using a microplate reader (Elx800, Bio-TEK, USA). Cell viability was expressed as a percentage relative to the control group.

**Wound healing assay**

For wound healing assays, MLE-12 cells were seeded into the wells at equal density and cultured to around 90% confluence to form a monolayer. A sterile 200 μL pipette tip was used to make vertical scratches across the monolayer. Detached cells were removed by washing twice with PBS, and fresh medium was added to each well. Images of the wounds were captured at 0 h to record the baseline wound area. Cells were then subjected to pharmacological interventions or plasmid transfection as indicated. Additional images were taken at 6, 12, and 24 h after wounding. The wound closure rate was quantified using ImageJ software as follows: Wound closure rate = (Initial wound area – Final wound area) / Initial wound area × 100%.

**Quantitative real-time PCR (RT-qPCR) analysis**

At the end of treatments, total RNA was extracted from cultured cells or lung tissues using RNAiso PLUS reagent (Takara Biotechnology, Dalian, China). RNA was reverse-transcribed into cDNA using the PrimeScript™ RT reagent kit (Takara Biotechnology, Dalian, China). Gene-specific primers (Sangon Biotech, Shanghai, China) are listed in Table 1. RT-qPCR was performed using a SYBR Green kit (Takara Biotechnology, Dalian, China) on a LightCycler 480 real-time PCR detection system (Roche, Switzerland) in triplicate wells for each target gene. Relative mRNA expression levels were normalized to *Actb* and calculated using the 2^–ΔΔCt^ method.

**Enzyme-linked immunosorbent assay (ELISA)**

The concentrations of TGF-β1 and IL-10 in mouse bronchoalveolar lavage fluid (BALF) and MLE-12 cell culture supernatants were measured using ELISA kits (Cat#EK0515, Cat#EK EK0411, Boster Biological Technology, Wuhan, China), according to the manufacturer’s instructions.

**Western blotting analysis**

Total protein was extracted using NP40 lysis buffer (Cat#P0013F, Beyotime, Shanghai, China) supplemented with protease inhibitor (Cat#P1005-1), phosphatase inhibitor (Cat#P1045-2), and PMSF (Cat#ST506). Lysates were centrifuged at 12,000 rpm for 30 min, and protein concentrations were determined using a BCA Protein Assay Kit (Cat#P0009, Beyotime, Shanghai, China). Equal amounts of protein (30-50 μg) were mixed with loading buffer, boiled at 100 °C for 10 min, separated on 10% SDS-PAGE gels, and transferred onto nitrocellulose membranes (Cat#PN66485, Pall, USA).

Membranes were blocked with 5% non-fat milk (Cat#A600669, SunnyBio, Shanghai, China) at room temperature for 2 h and then incubated overnight at 4 °C with the following primary antibodies (diluted according to the manufacturers’ instructions): anti-PINK1 (Cat#BC100-494, Novus, USA; 1:1000), anti-YAP1 (Cat#A1002, ABclonal, Wuhan, China; 1:2000), anti-phospho-YAP1 (Ser127, Cat#13008, CST, USA; 1:1000), anti-phospho-YAP1 (Ser397, Cat#13619, CST, USA; 1:1000), anti-AQP5 (Cat#ab78486, Abcam, UK; 1:1000), anti-SPC (Cat#ab211326, Abcam, UK; 1:1000), anti-Myc (Cat#16286-1-AP, Proteintech, USA; 1:5000), anti-Flag (Cat#20543-1-AP, Proteintech, USA; 1:20000), anti-GAPDH (Cat#60004-1-Ig, Proteintech, USA; 1:5000), anti-Histone H3 (Cat#4499, CST, USA; 1:1000) , anti-Parkin (Cat# 4211, CST, USA; 1:1000), anti-phospho-Parkin (Ser65, Cat#ab315376, Abcam, UK; 1:1000), anti-LC3 (Cat#18725-1-AP, Proteintech, USA; 1:1000), anti-P62 (Cat#ab56416, Abcam, UK; 1:1000), anti-TOM20 (Cat#11802-1-AP, Proteintech, USA; 1:5000), and anti-TIM23 (Cat#11123-1-AP, Proteintech, USA; 1:1000).

After washing, membranes were incubated with HRP-conjugated secondary antibodies (goat anti-mouse IgG, Cat#7076S, CST, USA, 1:10000; goat anti-rabbit IgG, Cat#111-035-003, Jackson ImmunoResearch, USA, 1:10000). Protein bands were visualized using an enhanced chemiluminescence system (ChemiDoc Touch, Bio-Rad, Canada), and band intensities were quantified with Image Lab software (Bio-Rad). GAPDH or Histone H3 was used as the internal control for normalization.

***In vitro* phosphorylation assay**

YAP1 protein was obtained via commercial approach (Cat#H00010413-P01, Abnova Corporation, China). Purified PhPINK1 was prepared according to the literature^5^ and kindly be gifted from Prof. Tang’s lab. YAP1 was incubated with *Ph*PINK1 at a 1:40 molar ratio in the buffer containing 20 mM HEPES pH 7.4, 150 mM NaCl, 5 mM MgCl2, 5 mM ATP, and 10 mM tris[2-carboxyethyl]phosphine) for overnight at 30°C. Phosphorylated YAP1 was purified by Superdex 200 Increase 10/300 GL (Cytiva). Western blotting confirmed phosphorylation using PINK1 antibody (Cat#BC100-494, Novus, USA; 1:1000), YAP1 antibody (Cat#A1002, ABclonal, Wuhan, China; 1:2000) and site-specific anti-phospho-YAP1 (Ser127, Cat#13008, CST, USA; 1:1000).

**RNA sequencing and bioinformatic analysis**

Total RNA was extracted from lung tissues of WT and *Pink1^-/-^* mice (n = 3 per group). Poly(A) RNA was purified from 1 μg of total RNA using Dynabeads Oligo (dT)25-61005 (Thermo Fisher, CA, USA), followed by fragmentation at 94 °C for 5-7 min using the Magnesium RNA Fragmentation Module (Cat#e6150, NEB, USA). The fragmented RNA was reverse-transcribed into cDNA, and the second strand was synthesized with DNA polymerase I (Cat#m0209, NEB, USA), RNase H (Cat#m0297, NEB, USA), and dUTP (Cat#R0133, Thermo Fisher, USA). After A-tailing, indexed adapters were ligated to both ends, and size selection was performed with AMPure XP beads. U-labeled second strands were removed with UDG (Cat#m0280, NEB, USA), and the libraries were amplified by PCR (95 °C for 3 min; 8 cycles of 98 °C for 15 s, 60 °C for 15 s, 72 °C for 30 s; final extension at 72 °C for 5 min). The average insert size was ~300 ± 50 bp. Paired-end sequencing (2 × 150 bp) was performed on the Illumina NovaSeq™ 6000 platform (LC-Bio, Hangzhou, China).

Raw reads were processed using fastp to remove adapters, low-quality bases, and undetermined nucleotides, and quality control was assessed simultaneously. Clean reads were aligned to the Mus musculus reference genome (GRCm38) using HISAT2, and assembled with StringTie. Transcriptome reconstruction was refined with gffcompare. Gene expression levels were quantified as FPKM using StringTie. Differentially expressed mRNAs were identified with the R package edgeR, with thresholds of fold change > 2 or < 0.5 and p < 0.05 based on nested linear model F-tests.

**Statistical Analysis**

Statistical analyses were performed using GraphPad Prism 9.0 (GraphPad Software Inc., USA). Data normality and homogeneity of variance were assessed using the D’Agostino–Pearson test and Brown–Forsythe test, respectively. For datasets meeting the assumptions of normal distribution and equal variances, statistical differences were evaluated using one-way analysis of variance (one-way ANOVA) or two-way analysis of variance (two-way ANOVA). For datasets that did not meet these assumptions, nonparametric tests (Kruskal–Wallis test) were applied. A P value < 0.05 was considered statistically significant.

**Code availability**

The computer codes to generate UMAP projection and Blended UMAP feature plot of single-cell RNA sequencing data within the article are accessible. Further inquiries can be directed to the corresponding author.

**References**

1.	Chen C, Gao TY, Yi HW, et al. Elevated ubiquitin phosphorylation by PINK1 contributes to proteasomal impairment and promotes neurodegeneration. *Elife.* 2025;14.

2.	Vaas M, Enzmann G, Perinat T, et al. Non-invasive near-infrared fluorescence imaging of the neutrophil response in a mouse model of transient cerebral ischaemia. *J Cereb Blood Flow Metab.* 2017;37(8):2833-2847.

3.	Saito M, Chen-Yoshikawa TF, Suetsugu K, et al. Pirfenidone alleviates lung ischemia-reperfusion injury in a rat model. *J Thorac Cardiovasc Surg.* 2019;158(1):289-296.

4.	Dong L, Liang F, Lou Z, et al. Necrostatin-1 Alleviates Lung Ischemia-Reperfusion Injury via Inhibiting Necroptosis and Apoptosis of Lung Epithelial Cells. *Cells.* 2022;11(19).

5.	Gao TY, Wang XZ, Xie YH, et al. PINK1 deficiency permits the development of Lewy body dementia with coexisting Abeta pathology. *Alzheimers Dement.* 2025;21(9):e70730.
