# Supplementary material for: PINK1 deficiency enhances alveolar regeneration and basement membrane remodeling by reducing YAP phosphorylation during lung ischemia-reperfusion injury
Source: Cell Death Dis. 2026 Aug 5;17(1):929. doi: 10.1038/s41419-026-09149-3 (PMC13724675; doi:10.1038/s41419-026-09149-3)
Supplement: Supplementary file 3 [file 41419_2026_9149_MOESM2_ESM.docx]

**Figure S1. AT2 cells expressing YAP also express PINK1 during lung ischemia-reperfusion injury.**

(A-B) UMAP projection of single-cell RNA sequencing data showing the expression pattern of *YAP1* (A) and *PINK1* (B) across cell populations^26^ (from GSE220797). (C) Blended UMAP feature plot showing the co-expression pattern of YAP1 and PINK1 across cell populations. (D) Schematic illustration of the left lung hilar clamping model. The left pulmonary hilum was occluded with a microvascular clamp for 1 h, followed by reperfusion. Mice were sacrificed at 0 h, 6 h, 24 h, 72 h, 7 d, and 14 d after reperfusion. (E) IL-10 concentration in BALF was determined using ELISA. Compared with the sham group: *p < 0.05, **p < 0.01, ***p < 0.001, ****p < 0.0001, one-way ANOVA. (F) Representative Western blotting of PINK1 in left lungs of mice with or without I1h/R3d modeling. (G) Representative immunofluorescence co-staining of SPC (green, AT2 cell marker) and PINK1 (red). White arrows indicate PINK1^+^SPC^+^ cells. (H) HE staining of left lungs. (I) IL-10 concentration in BALF was determined using ELISA. (J) Representative Western blotting analysis of Parkin, pho-Parkin, LC3, P62, TOM20, and TIM23 in the mouse lung. (K) Quantification of relative protein expression levels of Parkin, pho-Parkin, LC3-II/LC3-I, P62, TOM20, and TIM23. Comparisons were made as follows: vs. WT+sham group, *p < 0.05, **p < 0.01, ***p < 0.001, ****p < 0.0001, ns not significant; vs. WT+I/R group, #p < 0.05, ##p < 0.01, ###p < 0.001, ####p < 0.0001.

Data are presented as mean ± SD. One-way ANOVA was used for statistical analysis.

**Figure S2. Genetic manipulation on PINK1 and pharmacological modulation on YAP in mice.**

(A) Schematic of the experimental design: mice received intratracheal injection of AAV-PINK1shRNA or AAV-PINK1-cDNA for 3 weeks, followed by left lung I/R surgery. The left pulmonary hilum was clamped for 1 h and reperfused for 3 d before sample collection. (B) Mice received intratracheal injection of Flag-tagged AAV6 for 3 weeks. Representative immunofluorescence co-staining of SPC (green, AT2 cell marker) and Flag (red) in the lung slices. White arrows indicate Flag^+^SPC^+^ cells. (C) HE staining of mouse left lungs. (D) Schematic of experimental design: mice received intratracheal injection of AAV for 3 weeks, followed by left lung I/R surgery. Thirty minutes before surgery, mice were administered YAP inhibitor Verteporfin (VP, 100 mg/kg), YAP activator XMU-MP-1 (XMU, 1 mg/kg), or PBS via intraperitoneal injection. The left pulmonary hilum was clamped for 1 h and reperfused for 3 d before sample collection. (E) HE staining of mouse left lungs.

**Figure S3. Establishment of the** **Oxygen-Glucose Deprivation/Recovery model in MLE-12 cells.**

(A) Schematic illustration of the Oxygen-Glucose Deprivation/Recovery (OGD/R) protocol in MLE-12 cells. Cells were subjected to 4 h of oxygen-glucose deprivation followed by recovery to standard culture with complete medium under normoxic conditions(OGD 4h/R). (B) Cell viability of MLE-12 cells at different reperfusion time points after OGD/R. Data are presented as mean ± SD. *p < 0.05, **p < 0.01, ***p < 0.001, ****p < 0.0001, vs. Control, one-way ANOVA. (C) Representative immunofluorescence staining of Ki67 in MLE-12 cells at different reperfusion time points. (D) Quantification of the proportion of Ki67⁺ cells at different reperfusion time points. Data are presented as mean ± SD. *p < 0.05, **p < 0.01, ***p < 0.001, ****p < 0.0001, vs. the 1 h reperfusion group (1 h), one-way ANOVA.
